# Sulfonated, Disulfide‐Bridged Polymer Networks for Atmospheric Water Harvesting

**DOI:** 10.1002/smll.73271

**Published:** 2026-03-30

**Authors:** Joseph J. Dale, Paul Schweng, Mathilde Gerbaud, Robert T. Woodward

**Affiliations:** ^1^ Institute of Materials Chemistry and Research Faculty of Chemistry University of Vienna Vienna Austria; ^2^ Vienna Doctoral School in Chemistry University of Vienna Vienna Austria

## Abstract

Access to clean water is rapidly becoming a global crisis. The atmosphere holds up to six times the volume of water as all the rivers and lakes on earth combined, thus making atmospheric water harvesting a promising solution for clean water production. Sulfonated, hypercrosslinked‐style polymer networks are designed herein by Friedel–Crafts alkylation and thiol self‐condensation, using disulfide bonding to convey network mobility. Varying the amount of acid polymerization catalyst generates high‐capacity materials (1.39 g g^−1^water sorption at 90 % relative humidity (RH) and 25°C), capable of impressive adsorption (0.26 g g^−1^) at low humidities of 30%. The high adsorption capacity is predicted to be imparted by the rotational freedom of hydrophilic sulfonic acid groups, which accommodate significant water cluster formation and growth. We suggest that the polymers increase their own water harvesting capacity mechanically via conformational rearrangement, evidenced by an increased total uptake capacity of 1.60 g g^−1^ at 90% RH after repeated adsorption and desorption.

## Graphical abstract

Atmospheric water harvesting is a promising solution to the water crisis. Here, disulfide‐containing hyper‐crosslinked‐style polymers are prepared by combined Friedel–Crafts alkylation and thiol self‐condensation. Exhibiting exceptional uptake capacities, the polymers demonstrate dynamic bonding behavior. Predicted disulfide exchange, driven by water sorption cycling, alters the water uptake properties before relaxing to the original structural configuration under ambient conditions.

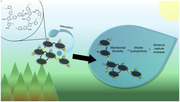

## 1 Introduction

Climate change, a growing population, and anthropogenic contamination are leading to a global clean water shortage [1]. Access to water impacts the available land on which humans live; it is no surprise that historical civilizations would choose rivers, lakes, and coastal regions on which to create their settlements. As these areas of natural clean water are consumed, population growth will require new sources of clean water. United Nations Sustainable Development Goal 6 states that we must “…achieve universal and equitable access…” to clean drinking water by the year 2030 [2]. The issue of water scarcity is gradually moving into the public eye, with droughts leading to poor irrigation on farmland and low crop yields set to impact livelihoods [3, 4]. Current methods of water collection are not without limitations. Fog harvesting is restricted to areas where fog can occur [5], while the desalination of non‐fresh water sources is energy‐intensive and generates large quantities of concentrated brine waste, making these processes undesirable [6]. The atmosphere holds up to 6× the liquid volume of water as all the lakes and rivers of the Earth combined [7]. Tapping the natural water reserves of the atmosphere is a logical next step in providing clean water to all.

Atmospheric water harvesting (AWH) materials adsorb moisture from the atmosphere passively [8]. A successful AWH material must be robust and easily synthesized, with a high capacity for water collection at a respectable rate [9]. These materials must also adapt to the natural conditions in which they are applied, as geographical location, season, and local urbanization can dramatically impact the relative humidity (RH). Efforts to apply functional porous materials to AWH are promising, with metal organic frameworks (MOFs) at the forefront of hygroscopic material design [10]. Indeed, Xu et al. have reported the successful implementation of MOFs for kilogram‐scale water harvesting [11], with MOF‐801 and MOF‐303 showing promise for water sorption at low RH. Hygroscopic salts have been added to MOF structures to enhance their AWH performance. Xu and coworkers report the LiCl‐loaded LiCl@MIL‐101(Cr) network, which exhibited 0.77 g g^−1^ adsorption at 30% RH and 30°C [12]. An et al. report the encapsulation of CaCl_2_ in MOF‐808 to yield a water uptake of 0.56 g g^−1^ at 30% RH and 25°C, translating to a water production rate of 1.8 kg kg^−1^ day^−1^ [13]. These materials, however, will always maintain the risk that metals may leach into the environment, which could then migrate into waterways, exacerbating the problem of clean water access further. The US Environmental Protection Agency has established an allowable lithium water content of 10 µg L^−1^ [14], while the World Health Organization (WHO) notes that calcium concentrations of up to 200 mg L^−1^ are rare in nature [15]. Covalent organic frameworks (COFs) are another branch of porous hygroscopic materials employed for AWH. Jiang et al. report the hydrazone‐linked Pythz‐COF that exhibited a water sorption of 0.83 g g^−1^ at 90% RH [16], while under the same conditions Chen et al. achieved an uptake of 0.64 g g^−1^ with their ketoenamine‐linked COF [17]. While certain MOFs and COFs face issues in their stability and reusability, with MOFs known to be water sensitive and undergoing hydrolysis upon exposure to moisture [18], leading MOFs and COFs in the AWH field exhibit water stability and promising uptake capacities. Factors such as coordination modes, ligand identities, and metal ion coordination are all under investigation to further improve the stability of MOFs [19]. Metal coordination is another promising route to AWH materials. An iron chloride‐loaded hydrogel reported by Guo et al. exhibited a water sorption capacity of 0.92 g g^−1^ (75% RH, 25°C) [20]. A NiCl_2_‐loaded gel reported by the same authors achieved the same capacity under the same conditions, with both gels reported applied beyond AWH to moisture energy harvesting [21].

Hypercrosslinked polymers (HCPs) are a collection of polymeric materials wherein the degree of crosslinking is such that a highly porous structure forms, providing an exceptional surface area [22]. Davankov reported the initial synthesis of a post‐synthetically crosslinked *poly*‐styrene, achieved by acid‐catalyzed Friedel–Crafts aromatic substitution [23]. The single‐step self‐condensation of chloromethyl [24], benzyl ether [25], and hydroxymethyl [26] monomers has since become a popular strategy for HCP design. Owing to their impressive surface areas, HCPs are considered for many applications, including hydrogen storage [27], CO_2_ capture [28], and heavy metal remediation [29]. Sulfonated HCPs (SHCPs) can be prepared using catalysts such as chlorosulfonic acid to confer hydrophilicity to the HCP structure for AWH, capturing up to 0.81 g g^−1^ at 90% RH and 25°C [30]. Combining a sulfonated network, impressive porosity, and a facile synthesis, these materials present a promising starting point for the design of AWH porous polymers.

**FIGURE 1:**
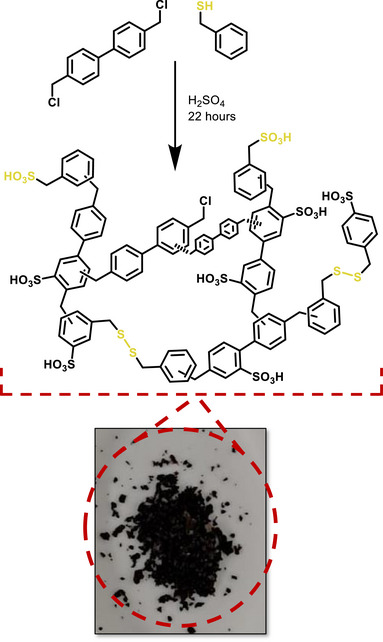
Representative reaction scheme showing the formation of hypercrosslinked polymers from BCMBP and BM, as prepared in this work, and a picture of the powder BM‐5.

Herein, we design largely non‐porous ‘HCP‐style’ polymer materials via the simultaneous self‐condensation of benzyl mercaptan (BM), the acid‐catalyzed Friedel–Crafts alkylation of 4,4‘‐bis(chloromethyl)‐1,1‘‐biphenyl (BCMBP), and the aromatic substitution of BM (Figure 1). Altering the catalyst‐to‐monomer ratios allows for the generation of materials with vastly differing water harvesting properties, with lower catalyst ratios providing low water harvesting capacities but fast adsorption–desorption cycling rates. High catalyst ratios confer high capacities and adsorption rates at low RH, but with much slower desorption rates, leading to a slower water harvesting potential. The structurally unrestricted nature of these materials allows for confirmational rearrangement upon adsorption, promoting a mechanically driven increase in water harvesting capacity. Disulfide HCPs have successfully been synthesized previously by Liu for the purpose of mercury adsorption from wastewater [31, 32]. However, we believe this is the first example of disulfide‐containing polymeric materials used for AWH.

## 2 Results and Discussion

BCMBP (0.50 g, 2.76 mmol) was dispersed in 4 mL 1,2‐dichloroethane (DCE) at room temperature under stirring. In a separate vessel, BM (0.15 g, 1.21 mmol) was mixed with 1 mL of DCE before being added to the BCMBP/DCE mixture. Sulfuric acid (95%) was then mixed with 1 mL DCE and added to the reaction flask in a 1:1, 3:1, or 5:1 molar ratio with BCMBP. The total DCE volume in each reaction was 6 mL. The mixture was heated to 80°C for 22 h, during which a dark orange solid formed that progressed to black within 3 h. After completion, the reaction was quenched with 20 mL of methanol, and the precipitate was filtered under vacuum before washing thrice on the filter with 50 mL of methanol. The obtained solid was then dried in an oven at 80°C for 24 h. Three samples were produced, labeled BM‐1, BM‐3, and BM‐5, representing the increasing catalyst ratios. The sulfuric acid acted as a dual Lewis acid catalyst/sulfonation agent, driving the polymerization of the BCMBP, and acting as an oxidizing agent to promote thiol self‐condensation. Network BM‐1 was a white/cream heterogeneous powder, BM‐3 was a fine brown homogeneous powder, and BM‐5 was a fluffy black homogeneous powder. Key reaction and product properties are detailed in Table 1. The yield of all samples was less than 50%, likely due to the use of sulfuric acid as a less efficient Friedel–Crafts catalyst than other Lewis acids, for example, aluminum chloride [33]. BM‐1 had a particularly low yield due to the limited quantity of catalyst available. Figures S1 and S2 show suggested mechanisms of Friedel–Crafts and thiol self‐condensation, respectively.

**TABLE 1:** Key properties of BM‐1, BM‐3, and BM‐5, including synthetic yields, char yields (a) determined using TGA, BET surface areas (b), sulfur content measured using CHNS elemental analysis (c), and water sorption (d) at 10%, 30%, and 90% RH derived from water sorption isotherm measurements.

Sample code	Catalyst equivalents	Yield (%)	Char yield (wt.%)^a^	BET Surface Area (m^2^ g^−1^)^b^	Sulfur content (wt.%)^c^	Water sorption (g g^−1^)^d^		
						10% RH	30% RH	90% RH
BM‐1	1	4.1 ± 1.9	0	0	5.65 ± 0.15	0.01	0.03	0.22
BM‐3	3	35.6 ± 9.1	18.4	26	14.29 ± 0.64	0.12	0.23	1.24
BM‐5	5	36.2 ± 11.1	29.7	41	17.88 ± 0.39	0.13	0.26	1.39

**FIGURE 2:**
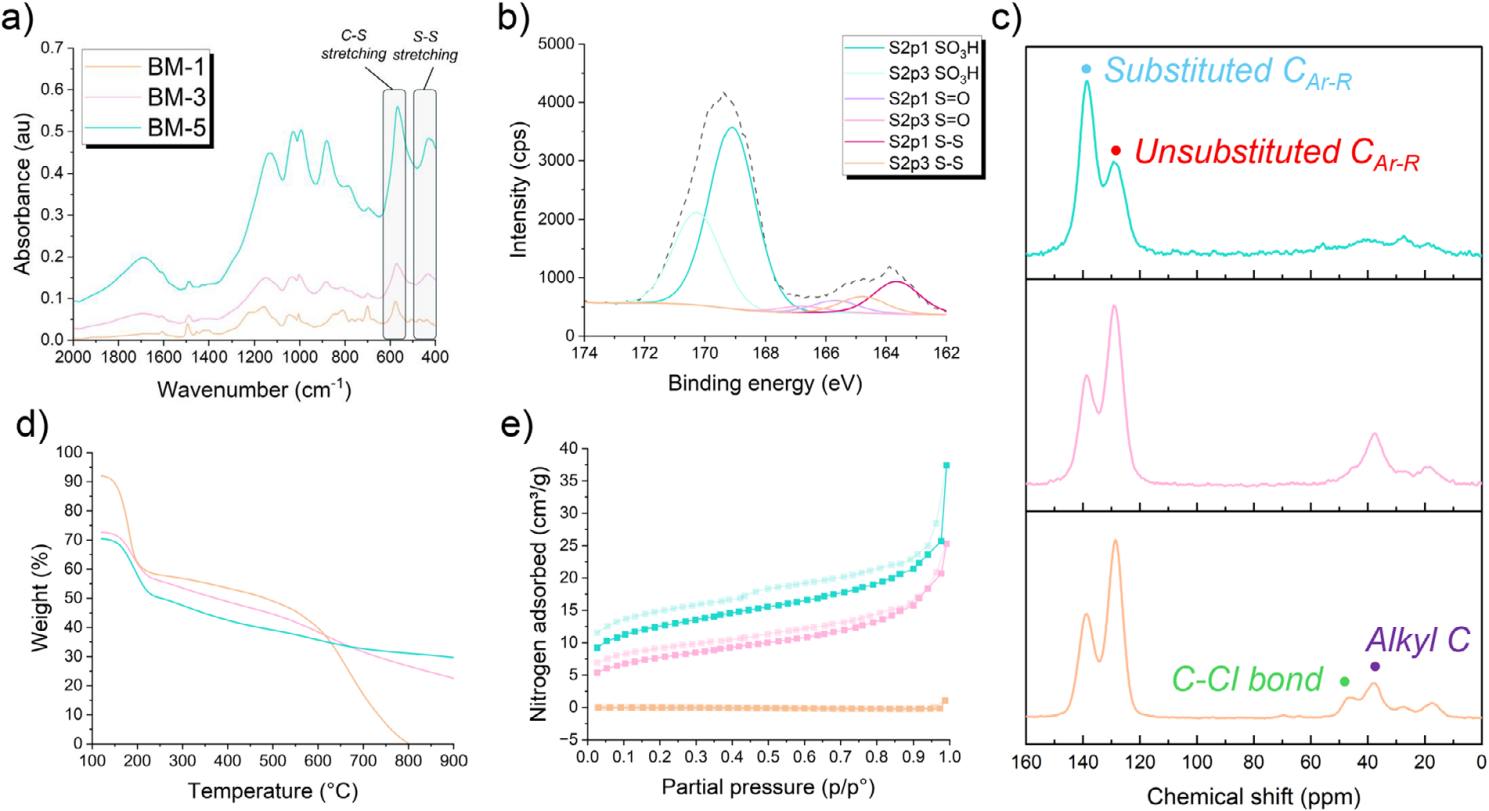
Analysis of BM‐1 (orange), BM‐3 (pink), and BM‐5 (teal). (a) FTIR analysis of the 2000–400 cm^−1^ region, highlighting the presence of C─S and S─S bonding. (b) Example high‐resolution S 2p XPS spectrum of BM‐3, showing S─S bonding from thiol‐coupling, C─S bonding from disulfide and sulfonic acid moieties, and S═O bonding from sulfonic acid groups. (c) Solid‐state ^13^C NMR showing residual C─Cl bonds are only present in BM‐1. (d) TGA thermograms demonstrating the complete decomposition of BM‐1 vs. the incomplete decomposition of the more crosslinked BM‐3 and BM‐5 networks. (e) N_2_ gas sorption analysis demonstrating uptake in BM‐3 and BM‐5, and no significant uptake in BM‐1.

A peak at 570 cm^−1^ in the Fourier transform infrared (FTIR) spectra (Figure 2a; Figures s3–s5) aligns with a C─S bond stretch present in all samples, suggesting successful sulfonation of aromatic positions and/or the retention of the C─S bond of BM. No thiol peaks were observed in any of the spectra, confirming complete condensation/oxidation of thiol groups. In BM‐3 and BM‐5, a peak at 434 cm^−1^ corresponding to the S─S bond stretch confirms the formation of disulfide linkages, absent in BM‐1. Higher ratios of sulfuric acid catalyst promote the self‐condensation of thiols to disulfides via a sulfenic acid intermediate and the elimination of water. The absence of an S─S peak in BM‐1 suggests that Friedel–Crafts alkylation is the more favored initial kinetic reaction, and that thiol self‐condensation occurs when more catalyst is present. Sulfonic acid S═O stretching is visible at 1160 cm^−1^ in BM‐1, 1150 cm^−1^ in BM‐3, and at 1130 cm^−1^ in BM‐5. The shift to a lower wavenumber with increasing catalyst ratio likely derives from a higher sulfonation density, leading to the retention of more water in the sample with improved hydrophilicity, and thus, hydrogen bonding causes a weakening of the S═O stretch. Water adsorption/retention is confirmed by the greater intensity of the O─H stretching in the 3000–3300 cm^−1^ region, as well as a broad peak at 1694 cm^−1^ attributed to a stretching vibration of water, observed to a greater intensity in the more sulfonated BM‐5 sample. The peak at 700 cm^−1^ is suggested to be unreacted C─Cl. X‐ray photoelectron spectroscopy (XPS) confirms the presence of S─S bonding in BM‐3 (Figure 2b), used here as a representative example. Peaks at 163.6 and 164.8 eV are ascribed to the S2p3/1 peak 2p1/2 peak of the S‐S bond, while peaks at 165.7 and 166.8 eV are assigned to the S═O sulfoxide/disulfone bonding (R‐SO‐S‐R) [34]. The peaks at binding energies of 169.1 and 170.3 eV are assigned to S 2p3/2 and S 2p1/2 S═O sulfonic acid bond, respectively [35]. We suggest, therefore, the oxidation of thiol groups to disulfide, sulfoxides/disulfones, and sulfonic acid groups during the course of the reaction.

Solid‐state ^13^C nuclear magnetic resonance (NMR) analysis (Figure 2c; Figures s6–s8) showed a decrease in the intensity of the alkyl peaks from BM‐1 to BM‐5, likely aligning with the formation of a more sulfur‐rich structure with increasing catalyst ratios. The electronegative, quadrupolar sulfur atoms cause a broadening of the alkyl peaks as the relaxation time lengthens [36]. A peak at 38 ppm (purple dot) is assigned to the alkyl C shift, while peaks at 27 and 17 ppm are designated as spinning sidebands. A peak at 47 ppm (green dot) is ascribed to the C─Cl bond, also observed in the FTIR spectra, which also decreased from BM‐1 to BM‐5 due to enhanced Friedel–Crafts alkylation. The peaks at 128 ppm (red dot) and 139 ppm (blue dot) correspond to the unsubstituted and substituted aromatic C─C bonds, demonstrating a higher degree of aromatic substitution and therefore sulfonation in network BM‐5. It is also suggested that a Pummerer rearrangement of thiols with BCMBP may occur (Figure S9), adding to a more complex bonding structure, however it is difficult to confirm due to the resolution in the alkyl region of the spectra.

Thermogravimetric analysis (TGA) under a N_2_ atmosphere (Figure 2d) showed the release of weakly bound water during a 30 min isothermal hold at 120°C, while more strongly bound water was released upon further heating; a decomposition temperature range of 160.0°C–165.5°C was observed. In BM‐1, 3, and 5, a mass loss of 41.9%, 43.6%, and 51.1%, respectively, was attributed to adsorbed water. Network BM‐1 decomposed completely by 800°C, while BM‐3 and BM‐5 presented char yields of 18.4 wt.% and 29.7 wt.%, respectively. The increased stability of BM‐3 and BM‐5 is ascribed to a greater degree of crosslinking, again due to increased catalyst ratios. Differential scanning calorimetry (DSC) showed no glass transition temperature in any of the samples (Figures s10–s12), characteristic of HCP networks. A mild endothermic peak at 96°C is ascribed to the release and evaporation of water.

Elemental analysis (Table S1) of dry samples yielded a sulfur content of 5.65 ± 0.15 wt.% in BM‐1, 14.29 ± 0.64 wt.% in BM‐3, and 17.88 ± 0.39 wt.% in BM‐5. N_2_ gas sorption analysis at 77 K (Figure 2e) showed no measurable adsorption in BM‐1 and type II isotherms for BM‐3 and BM‐5. No significant Brunauer–Emmett–Teller surface area (BET_SA_) was calculated for BM‐1, while BM‐3 and BM‐5 exhibited a BET_SA_ of 26 and 41 m^2^ g^−1^ respectively. The greater catalyst ratio used in BM‐5 led to a greater crosslinking degree, resulting in an increased BET_SA_. Hysteresis looping becomes more prominent with increasing catalyst ratio, aligning with the increased porosity.

**FIGURE 3:**
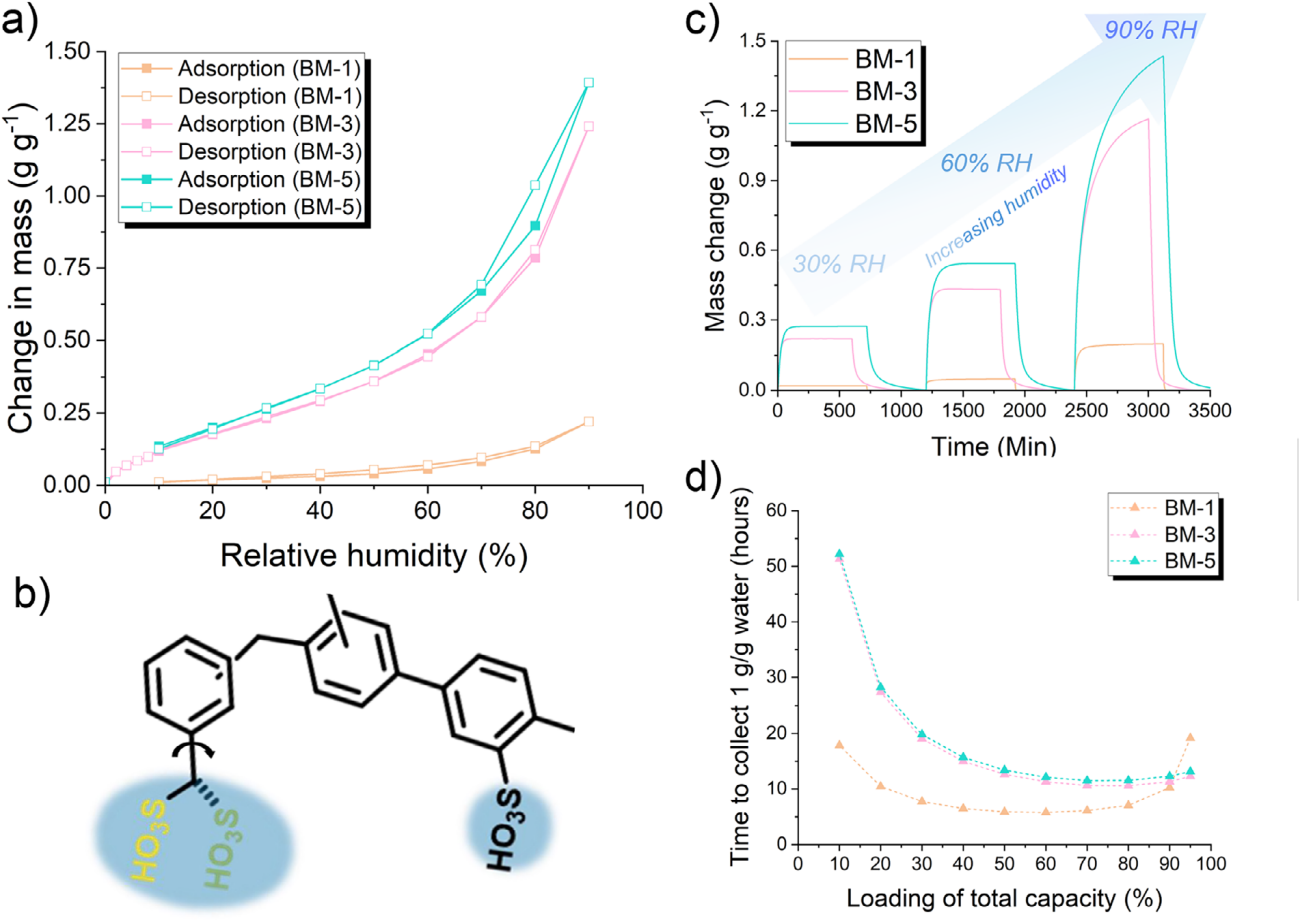
(a) Water adsorption isotherms of BM‐1, BM‐3, and BM‐5 at 25°C, demonstrating the increasing adsorption properties with increasing catalyst ratio. (b) Schematic representation of the mobile hydrophilicity conferred by BM. In this case, the sulfonic acid group has rotational freedom, increasing the space in which water clusters may form and grow. (c) Water sorption profiles of all samples at 30%, 60%, and 90% RH. Adsorption and desorption were each conducted for 12 h. (d) The theoretical time required for each material to adsorb 1 g g^−1^ of water at 90% RH. Adsorption and desorption rates measured in Figure 3c are used to calculate the time required to load the material to various percentages of its total capacity, before complete desorption. Polymer BM‐1 can theoretically collect 1 g g^−1^ in 5.8 h after loading to 60% capacity, while both BM‐3 and BM‐5 would collect the same amount after 10.6 and 11.5 h, respectively, after loading to 70% capacity.

The water sorption isotherms of the networks were collected using dynamic vapor sorption (DVS) at 25°C (Figure 3a), unless otherwise specified. The maximum sorption capacities for BM‐1, BM‐3, and BM‐5 at 90% RH were 0.22, 1.24, and 1.39 g g^−1^, respectively. Considering the theoretical work of Shih et al. on non‐S‐shaped (N‐S) isotherms, the following mechanism and structure–property relationships are proposed [37]. Increasing the catalyst ratio increased the degree of sulfonation and incidence of thiol oxidation, creating highly hydrophilic nucleation sites that bind water at low RH [38], consistent with N‐S(I)‐type behavior. At intermediate RH, additional water molecules bind to these initial sites, leading to cluster formation and giving rise to the gradual uptake typical of N‐S(II). The introduction of network mobility and rotational freedom of sulfonic acid groups in BM‐5 allows for further cluster growth and eventually pore condensation in the small degree of porosity exhibited, as shown by the steep rise in uptake at high RH. Therefore, a gradual shift from surface‐controlled adsorption at low RH, to uptake dominated by water–water interactions at high RH is proposed. Importantly, at low RHs such as 10% and 30%, BM‐5 was able to adsorb 0.13 and 0.26 g g^−1^, respectively, while BM‐3 was able to adsorb 0.11 and 0.22 g g^−1^ under the same conditions. Adsorption at low RH is of critical importance if materials such as these are to be applied for AWH in arid regions. No significant adsorption was observed at low RH for BM‐1, confirming that high hydrophilicity is key for water cluster seeding and growth at low RH. The time taken for each sample to adsorb 0.20 g g^−1^ mass of water at 90% RH and 25°C was determined to be 483.3 min (BM‐1), 8.4 min (BM‐3), and 9.3 min (BM‐5). The water diffusivity, derived from Fick's diffusion law, was estimated to be 1.5 × 10^−10^ m^2^ s^−1^ at 30% RH, consistent with the expected adsorption properties of polymeric adsorbents [39].

The theoretical cycling rate of each network was calculated, determining the capacity the material should adsorb to, before complete desorption, for optimum water yields (Figure 3d; Figure S13 and Tables S2–S5). BM‐1 demonstrated a theoretical time to adsorb 1 g g^−1^ of water at 90% RH and 25°C of 5.8 h, adsorbing to 60% maximum capacity before complete desorption, yielding a theoretical water collection rate of 4.1 L kg^−1^ day^−1^. For comparison, under the same conditions, BM‐5 exhibited a theoretical time to adsorb 1 g g^−1^ of water of 11.5 h, thus a collection rate of 2.0 L kg^−1^ day^−1^. The desorption rate of BM‐1 within this capacity region is faster than that of BM‐5; complete desorption of water clusters requires a longer time due to the greater sulfonation degree, increasing the hydrophilicity and attracting the water clusters more strongly. BM‐1 has a low overall capacity, but water removal upon desorption is fast, as the lower sulfonation degree leads to primarily surface water adsorption rather than cluster formation and growth. Therefore, adsorption–desorption cycling is quicker than BM‐5, where the capacity is greater, but the desorption profile is longer in the low water capacity region. If desorption to completion is not required, then it could be predicted that the greater capacity of BM‐5 would promote a much greater collection rate than BM‐1, with desorption occurring rapidly at high water capacities as condensed surface water is removed. It must be noted that at low RH values, no material prevails, with all networks cycling to a similar degree.

**FIGURE 4:**
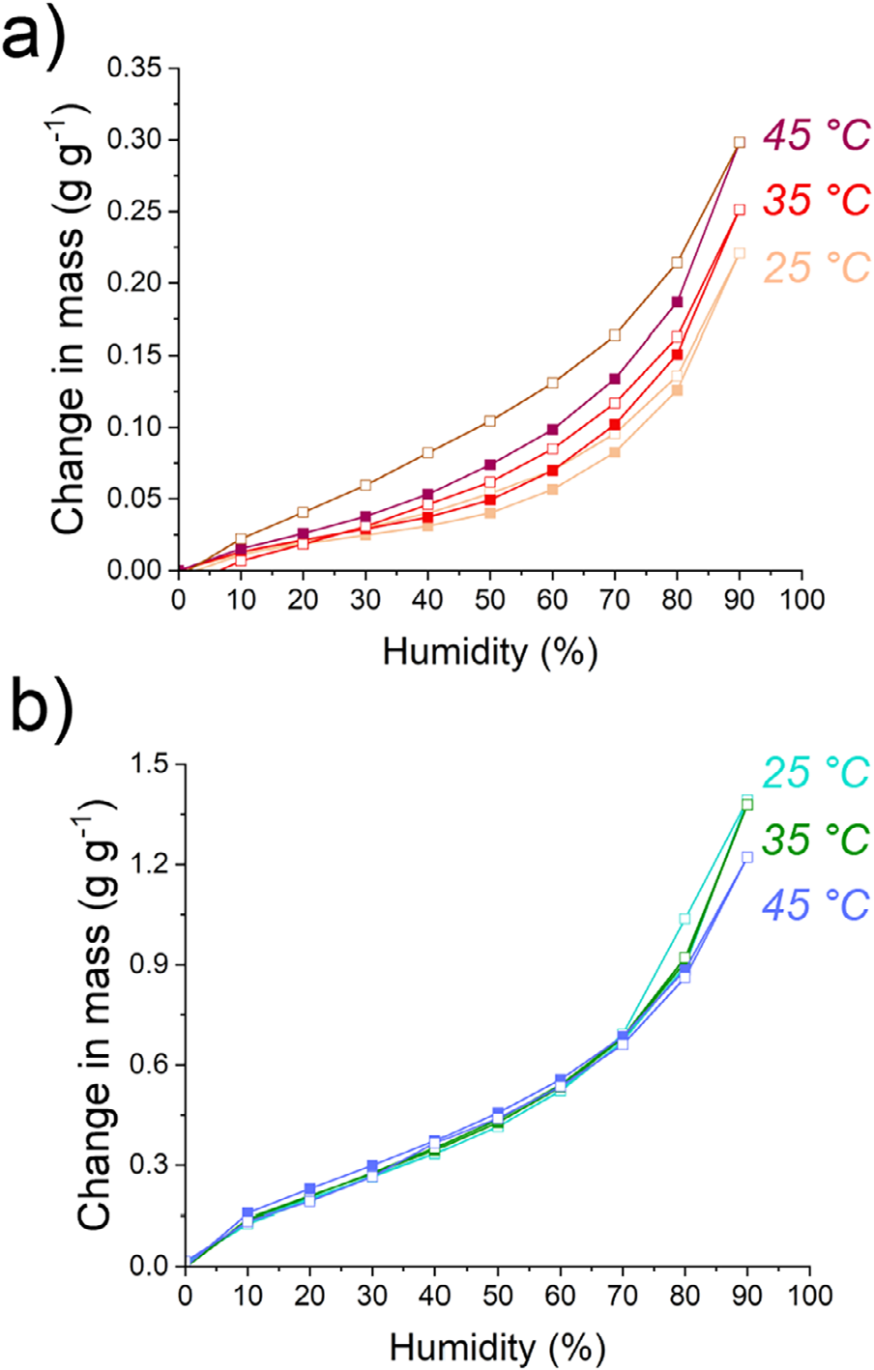
The isotherms applied in the calculation of the isoteric heat of adsorption. (a) BM‐1, demonstrating an increase in the isotherm adsorption maximum with increasing temperature. (b) BM‐5, demonstrating a slight decrease in the isotherm adsorption maximum with increasing temperature.

The isoteric heat of adsorption (ΔH_Ads_) for BM‐1 (Figure 4a) and BM‐5 (Figure 4b) was calculated using the Clausius–Clapeyron equation, applying isotherms at 25°C, 35°C, and 45°C. ΔH_Ads_ values of Q_st_ = 40 and 45 kJ mol^−1^ were determined for BM‐1 and 5, respectively, similar to that of bulk water at Q_st_ = 44 kJ mol^−1^ [40]. The greater ΔH_Ads_ for BM‐5 alludes to the higher interaction energy of water with the material, and thus the increased hydrophilicity conferred by a more densely sulfonated network within the material. Comparing the adsorption isotherms at increasing temperature, an increase in hysteresis was observed in BM‐1, with a growth in the adsorption maximum. Contrarily, BM‐5 exhibited a decrease in adsorption maxima and a loss of hysteresis, suggesting that at elevated temperatures, a structural rearrangement may lead to a loss of porosity. Complete desorption via humidity swing from maximum capacity (reduction from 90% to 0% RH) took 143.5 min at 45°C compared to 550.3 min at 25°C, thus demonstrating the superior desorption rates at higher temperatures. The g g^−1^ collection rates at 25°C and 45°C are shown in Figure S14. Desorption was measured by conditioning BM‐5 at 75% RH and room temperature (variable between 20°C and 23°C) for 3 days before heating the samples to various temperatures (25°C, 40°C, 55°C, and 70°C) on the TGA and monitoring their mass loss over 1 h (Figure S15). Elevated temperatures accelerated water desorption, leading to the removal of most of the adsorbed water within 20 min at 70°C. A lower mass loss is recorded than would be expected based on the measured isotherm, likely due to the air flow of the TGA instrument removing adsorbed water while initiating the measurement.

**FIGURE 5:**
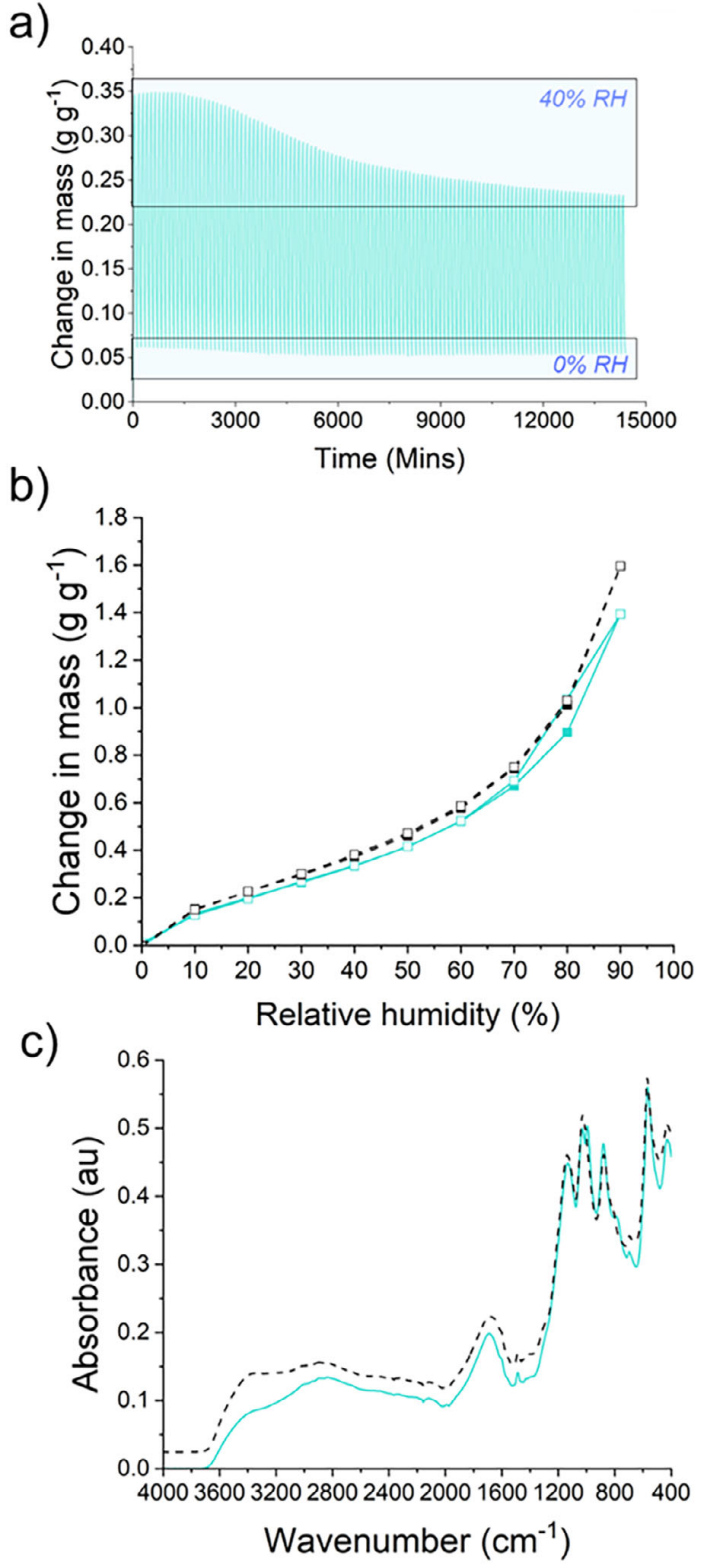
(a) Cycling of BM‐5 between 0% and 40% RH over 60 cycles. One cycle comprised of 1 h uptake and 1 h desorption. (b) Water sorption isotherms of BM‐5 before (green) and after (black) cycling, showing an increase in maximum capacity from 1.39 up to 1.60 g g^−1^. (c) FTIR analysis pre‐ and post‐cycling, showing no chemical alteration to polymer structure after 60 cycles.

The stability of BM‐5 was tested across 120 water adsorption–desorption cycles, in which adsorption occurred at 40% RH and desorption at 0% RH for 1 h at each RH (Figure 5a). The BM‐5 network showed a general decrease in adsorption capacity while held for 1 h at 40% RH. After cycle 1, the adsorption capacity was 0.33 g g^−1^, while after the final cycle, the capacity had decreased to 0.25 g g^−1^. The desorption capacity remained largely the same with each cycle. A water sorption isotherm measured on the material after this cycling showed an increase in the maximum capacity from 1.39 to 1.60 g g^−1^, with a general rise in sorption capacity at all values of RH (Figure 5b). At each value of RH, the mass is allowed to stabilize before increasing the RH further. S─S bonds are well known to be dynamic, contributing to living polymer systems and covalent adaptive networks [41]. Residual thiols in the system may form nucleophilic S^−^ species upon exposure to water [42, 43], instigating disulfide exchange and altering the material's porous properties. Post‐cycling, BM‐5 was dried for 24 h at room temperature before cycling again under the same conditions. Interestingly, BM‐5 recovered its pre‐cycling uptake capacity (Figure S16), suggesting that the system reverts to its original state as the equilibrium relaxes to the former configuration. It is tentatively posited that the disulfide bonds confer a dynamic structure upon water sorption. FTIR analysis conducted after cycling confirmed no measurable chemical alteration in the material structure after cycling (Figure 5c). BM‐5 also proved stable when cycling between 0% and 90% RH, achieving uptakes of >0.8 g g^−1^ during each 1 h cycle (Figure S17).

AWH in BM‐5 is suggested; therefore, to progress through different mechanisms. First, disulfide exchange, initiated by consistent moisture exposure, leads to a structural rearrangement during adsorption, granting a dynamic structure. Second, we performed a swelling study on the BM‐X series (Table S6). The extent of swelling increases from BM‐1 to BM‐5, mirroring the trend observed in water uptake. However, the magnitude of swelling does not quantitatively match the increasing adsorption capacities, indicating that while polymer network swelling likely facilitates water uptake, additional structural factors must also contribute. Thus, we suggest that enhanced cluster formation/growth is also a dominant water uptake mechanism, driven by the high density of hydrophilic groups, for example, sulfonic acid, disulfone bonding. Finally, we tentatively suggest that, in some cases, the thiol groups on the BM moiety may oxidize to sulfonic acid groups. The resulting methanesulfonic acid groups may possess more rotational freedom than the sulfonic acid groups produced by direct sulfonation of the aromatic rings, providing larger hydrophilic spaces within the polymers (Figure 3b). However, we aim to investigate and understand this theory in our future work.

## 3 Conclusions

‘HCP‐style’ networks were synthesized via the Friedel–Crafts aromatic substitution of BCMBP and BM, and the acid‐catalyzed thiol self‐condensation of BM. Three materials were prepared using increasing catalyst loadings relative to the aromatic monomers. A lower catalyst loading prepared a material with a water uptake capacity of 0.22 g g^−1 ^wt.% at 90% RH, theoretically able to cycle quickly and collect its own weight in water within 5.8 h. A higher catalyst ratio generated materials with a water uptake capacity of up to 1.39 g g^−1^ at 90% RH, and even 0.13 and 0.26 g g^−1^ at 10% and 30% RH, respectively. We posit that mobile hydrophilic methanesulfonic acid moieties, coupled with a mobile disulfide network, allow for high water uptake capacities as the material can form an increased number of water clusters. We predict a structural rearrangement of network BM‐5 upon cycling, thus allowing for an increase in adsorption capacity to 1.60 g g^−1^. The high absolute capacity of BM‐5, coupled with the temperature‐dependent desorption kinetics, may promote a benefit in alternative AWH applications, such as extended soil irrigation. In the desire for greater understanding of water harvesting mechanisms, we propose that non‐porous polymers could offer a promising new branch of attractive water sorption properties.

## Author Contributions

**Joseph J. Dale**: Writing – review & editing, Writing – original draft, Methodology, Investigation, Formal analysis, Data curation, Funding acquisition, Conceptualization. **Paul Schweng**: Formal analysis, Writing – review & editing. **Mathilde Gerbaud**: Investigation, Writing – review & editing. **Robert T. Woodward**: Writing – review & editing, Methodology, Funding acquisition, Conceptualization.

## Conflicts of Interest

None of the authors have a conflict of interest to disclose.

## Supporting information




**Supporting File**: smll73271‐sup‐0001‐SuppMat.docx.

## Data Availability

The data that supports the findings of this study are available in the supplementary material of this article.
