# Supplementary material for: Sulfonated, Disulfide‐Bridged Polymer Networks for Atmospheric Water Harvesting
Source: Small. 2026 Mar 30;22(29):e73271. doi: 10.1002/smll.73271 (PMC13206371; doi:10.1002/smll.73271)
Supplement: Supplementary file 1 — Supporting File: smll73271‐sup‐0001‐SuppMat.docx. [file SMLL-22-e73271-s001.docx]

Sulfonated, Disulfide-Bridged Polymer Networks for Atmospheric Water Harvesting

Joseph J. Dale,^1, *^ Paul Schweng,^1,2^ Mathilde Gerbaud,^1^ Robert T Woodward^1, *^

^1^ Institute of Materials Chemistry and Research, Faculty of Chemistry, University of Vienna, Währinger Straße 42, 1090, Vienna, Austria

^2^ Vienna Doctoral School in Chemistry, University of Vienna, Währinger Straße 42, 1090, Vienna, Austria

* Email: [Joseph.dale@univie.ac.at](mailto:Joseph.dale@univie.ac.at), [robert.woodward@univie.ac.at](mailto:robert.woodward@univie.ac.at)

**Materials**

All chemicals were purchased and used without further purification. 4,4‘-Bis(chloromethyl)-1,1‘-biphenyl (BCMBP, 95%), 1,2-dichloroethane (DCE, >99.0%), and methanol (>99.8%) were purchased from Sigma Aldrich. Benzyl mercaptan (BM, >96%) was purchased from Tokyo Chemical Industry. Sulfuric acid (>95%) was purchased from Fischer Scientific.

**Characterization**

**Fourier transform infrared (FTIR) spectroscopy**

FTIR analysis was conducted using a Bruker Tensor II Spectrometer. 64 scans were conducted between 4000-400 cm^-1^ at a resolution of 4 cm^-1^.

**X-ray photoelectron spectroscopy (XPS)**

XPS was conducted using a Nexsa Photoelectron Spectrometer (Thermo Scientific). All measurements were performed using Al-Kα X-rays and a spot size of 400 µm. Evaluation of the spectra was performed using Avantage software (v5.9931, Thermo Fisher Scientific).

**Elemental analysis (CHNS)**

Elemental analysis was performed using a Eurovector EA 3000 CHNS-O Elemental Analyser. 1-2 mg of sample was weighed into tin vials (4×6 mm) and measured in triplicate. 1000 °C combustion temperature and 750 °C reduction temperature were applied in helium (99.999+) carrier gas.

**Solid state ^13^C nuclear magnetic resonance (ssNMR)**

ssNMR spectra were collected using a Bruker AV III NEO 500 wide bore system (Bruker BioSpin, Rheinstetten, Germany) 600 MHz NMR spectrometer using a 4 mm triple resonance magic angle spinning probe. ssNMR was conducted between 15 - 25 mg of material was packed into a 4 mm zirconia CRAMPS rotor. The 13C NMR resonance frequency was 125.78 MHz, and the MAS rotor was set to spin at 14 kHz. Cross polarisation was achieved by a ramped contact pulse using a contact time of 3 ms. During acquisition 1H was high power decoupled using SPINAL with 64 phase permutations. The 1H π/2 pulse was 2.5 µs, the relaxation delay was set to 4 s. Approximately 2000 scans were required to achieve a sufficient signal to noise. The chemical shifts for 13C are reported in ppm and are referenced external to adamantane by setting the low field signal to 38.48 ppm.

**Differential scanning calorimetry (DSC)**

DSC was performed using a TA Instruments Discovery series operating a heat-cool-heat cycle. In each analysis, the temperature was equilibrated at 20 °C for 1 minute, before heating to 200 °C at 10 °C min^-1^, cooling to -50 °C at 10 °C min^-1^ and finally heating to 200 °C at 10 °C min^-1^ under nitrogen. Upon reaching each target temperature, a 1 min isothermal was applied. Samples were prepared in sealed Tzero aluminium pans.

**Thermogravimetric analysis (TGA)**

TGA was performed on a TA Instruments Discovery TGA. All samples were heated at 10 °C min^-1^ to 120 °C before a 30 min isothermal was applied to dry the samples. After this a 10 °C min^-1^ heating rate was applied to a maximum of 900 °C under nitrogen at a flow rate of 25 mL min^-1^.

**N_2_ gas sorption**

Nitrogen adsorption-desorption isotherms were recorded at –196 °C (77 K) using a TriStar II (Micromeritics Instrument Corporation). Samples were degassed for 24 h at 120 °C under N_2_ atmosphere using a FlowPrep 060 (Micromeritics Instrument Corporation). Sample surface areas were calculated using the Brunauer-Emmett-Teller (BET) method on the adsorption branch between 0.05 - 0.2 P/P0.

**Dynamic vapour sorption (DVS)**

DVS was conducted using a DVS resolution (Surface Measurement Systems). Approximately 10-20 mg of each sample was weighed into an aluminium pan and placed in an atmosphere of DI water. For isotherm measurements the RH was increased in 10 % step increments up to 90 % RH then decreased in the desorption curve. An 8-12 hour drying step at 0 % RH was implemented. This differed between samples, but all were dry by 8 hours. All measurements were conducted at 25 °C.

Experiments were conducted to investigate the rates of water adsorption and desorption. The materials were exposed to 30%, 60%, and 90% RH for 12 hours (Figure 3c). The samples were then exposed to 0% RH for 12 hours to recondition and measure the desorption rate. All analysis was conducted at 25 °C. The theoretical time taken for each material to collect 1 g of water per g of material was calculated in accordance with equation 1 below (see SI for derivation).

$\left( S1 \right) Time taken to collect 1 g of water=\frac{100}{\% mass of water} x$($t_{Ads}+t_{Des})$

Time of adsorption was determined as the time taken to reach a percentage of the total capacity at a designated RH e.g. time taken to reach 95% total adsorption capacity at 30% RH. Time of desorption was determined from the point at which the % total capacity reached 0 in the desorption curve, starting from the relevant capacity e.g. time taken to reach 0% from 95% of the total capacity. The calculated values are recorded in Table S2.

DVS rate measurements: Equation 1 derivation

(S2) $Mass of water per cycle (M_{Cycle})=\frac{\% mass of water}{100}$

(S3) $Cycle time (t_{cycle})=t_{Ads}+t_{Des}$

(S4) $Number of cycles to collect 1 g water (C)=\frac{1}{M_{Cycle}}$

(S5) $Time taken to collect 1 g of water=C x t_{cycle}$


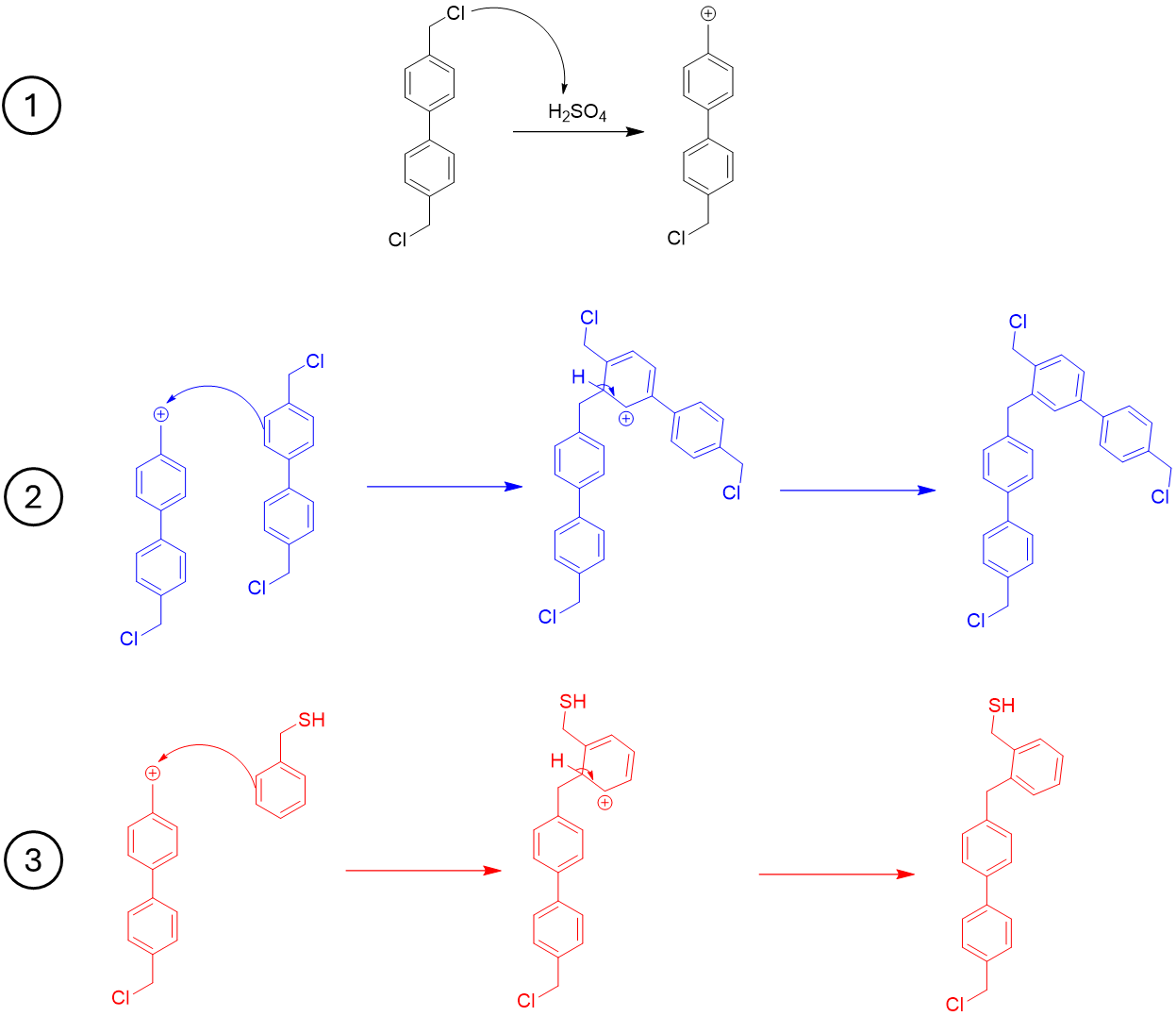


Figure S1. Friedel-Crafts aromatic substitution mechanism of BCBP with BCBP (blue) and BCBP with BM (red).

Figure S2. Thiol self-condensation mechanism *via* a sulfenic acid intermediate to form a disulfide bridge.


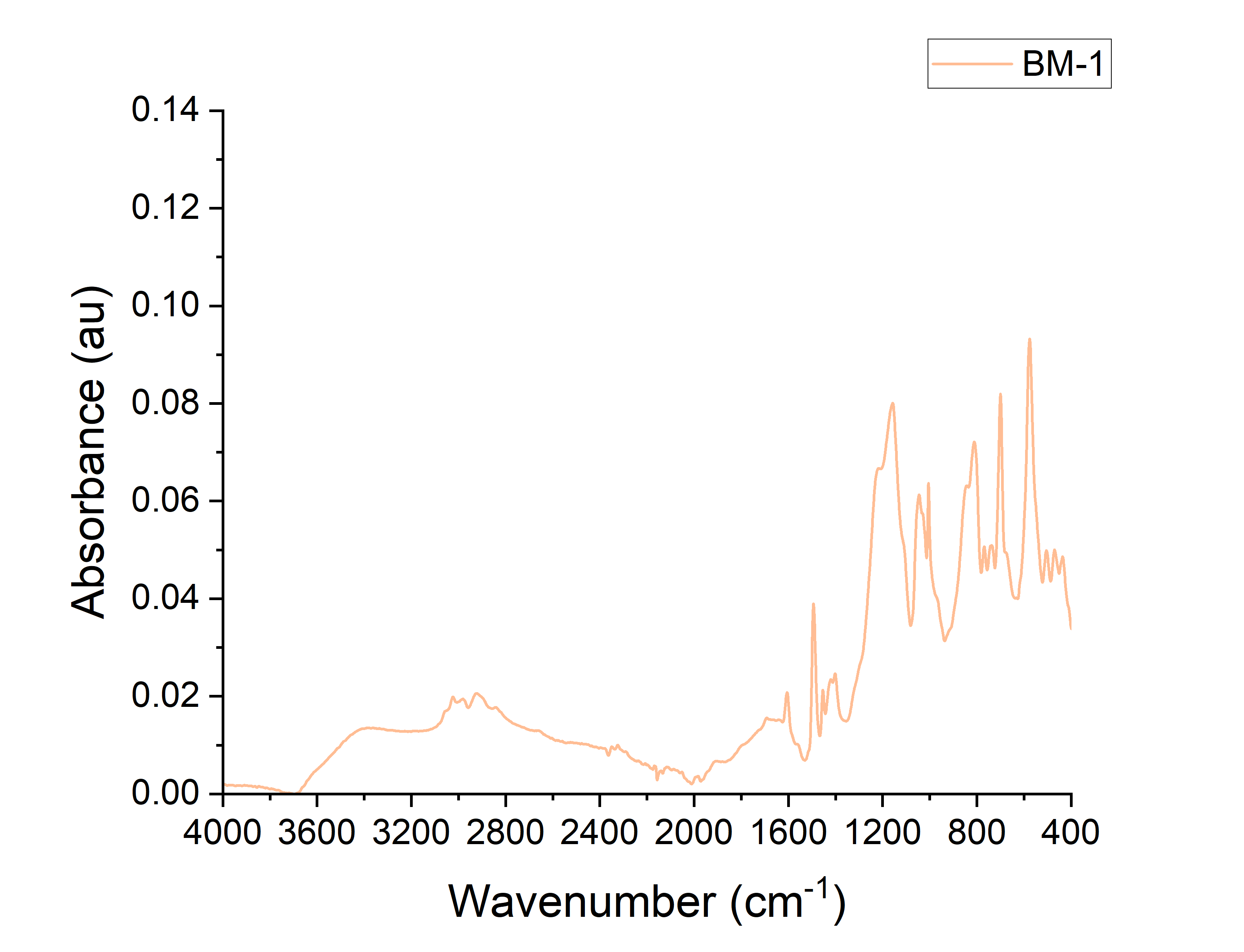


Figure S3. Full spectrum FTIR analysis of BM-1, scanning between 4000-400 cm^-1^.


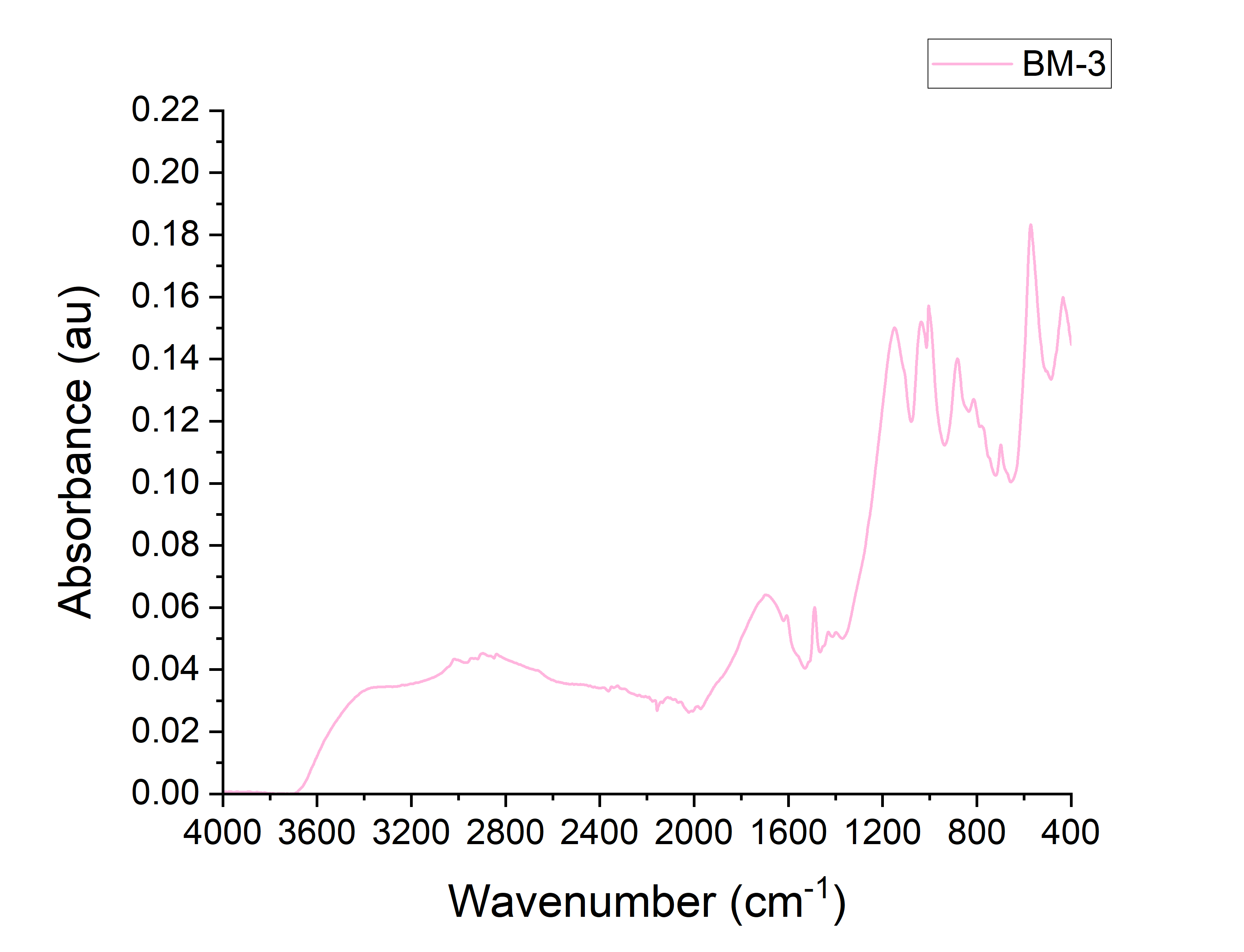


Figure S4. Full spectrum FTIR analysis of BM-3, scanning between 4000-400 cm^-1^.


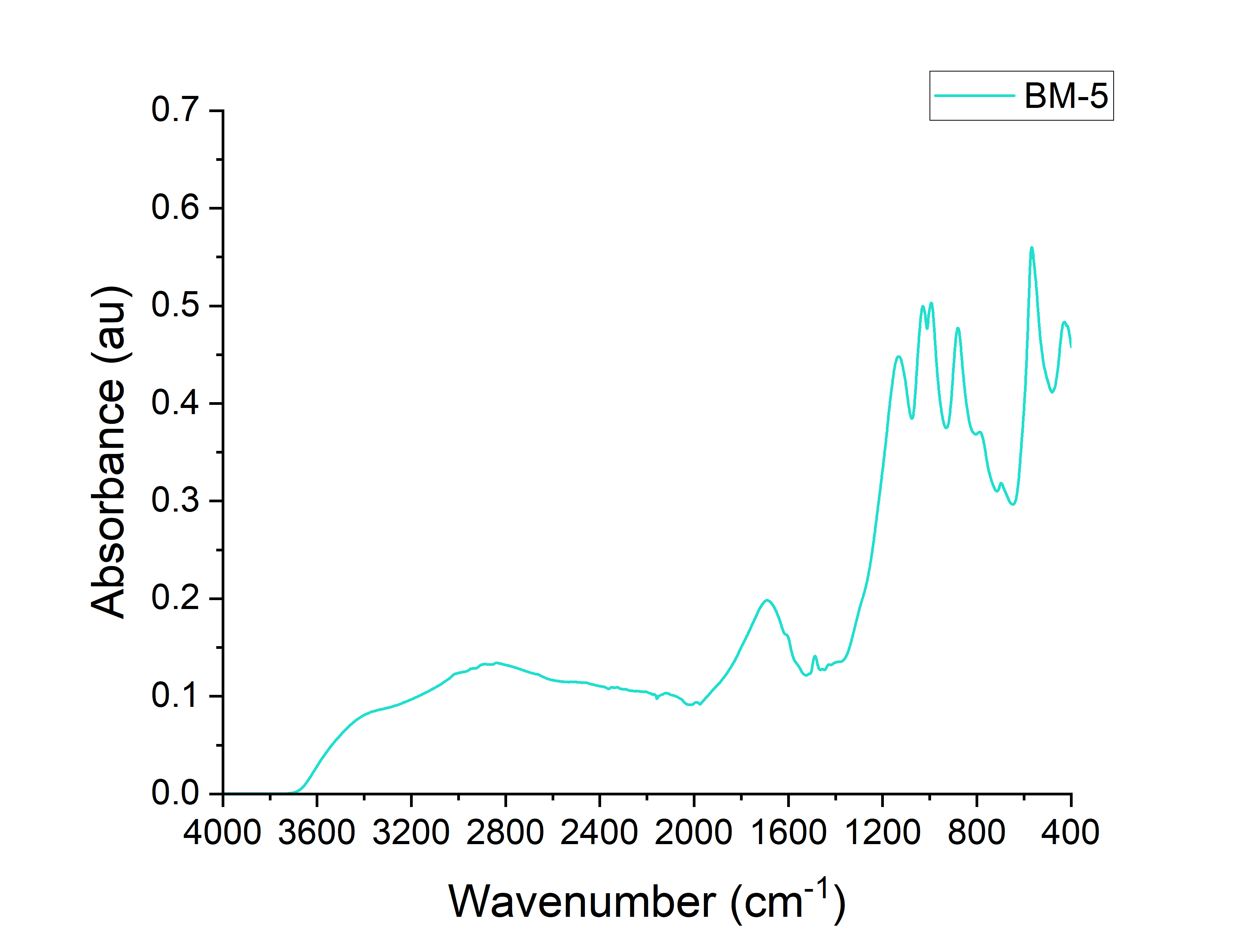


Figure S5. Full spectrum FTIR analysis of BM-5, scanning between 4000-400 cm^-1^.


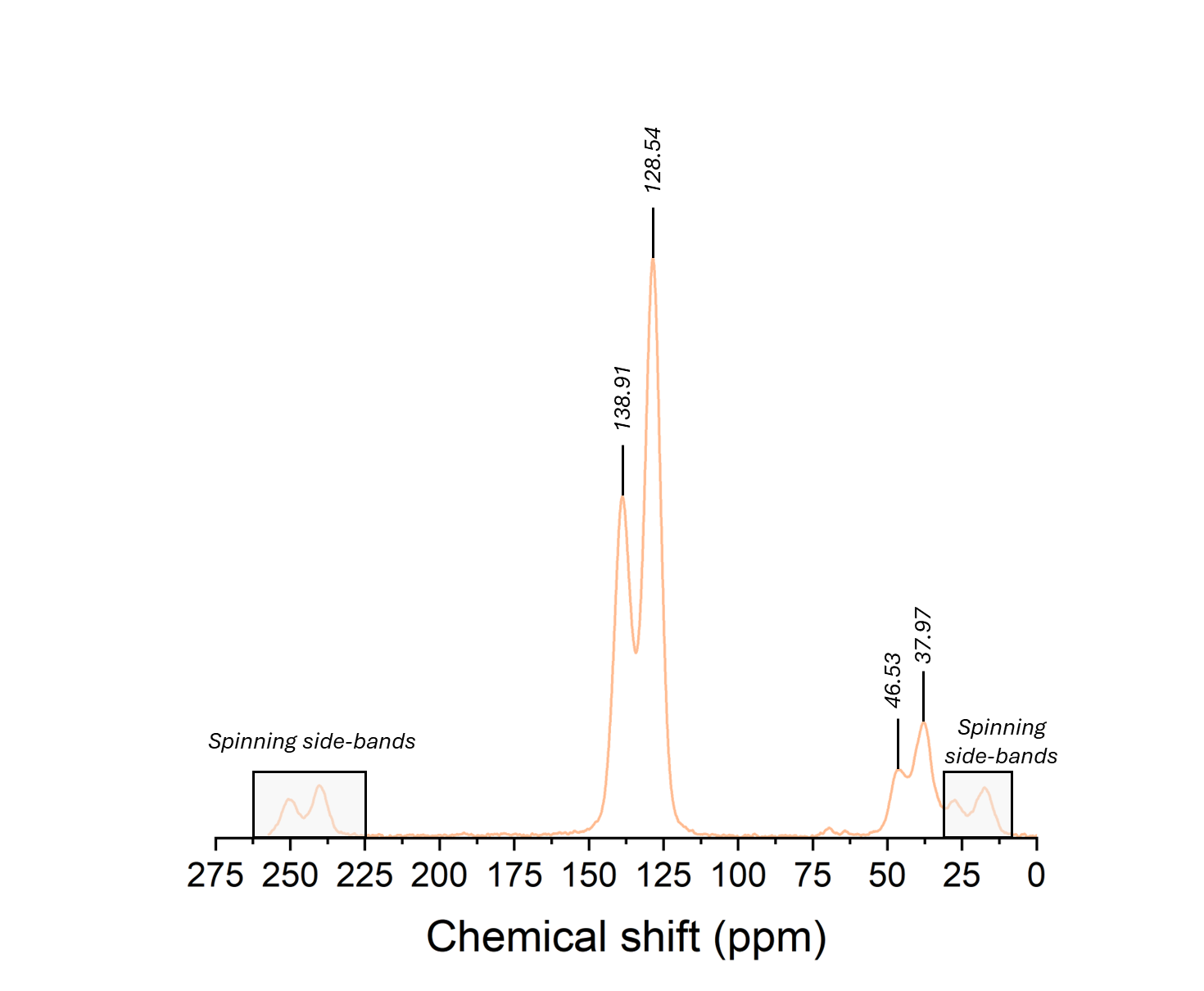


Figure S6. ^13^C NMR of BM-1, expanded to show spinning sidebands.


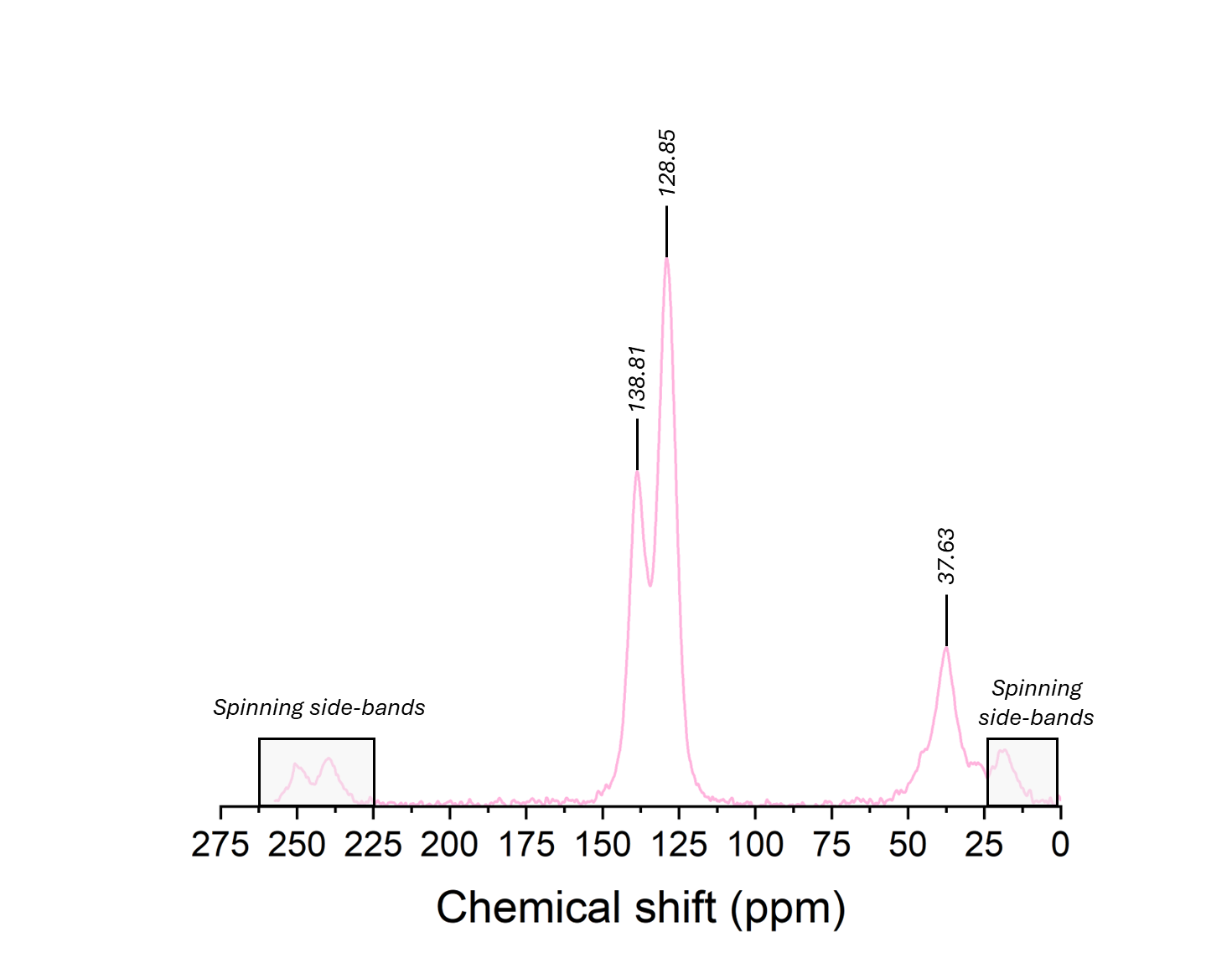


Figure S7. ^13^C NMR of BM-3, expanded to show spinning sidebands.


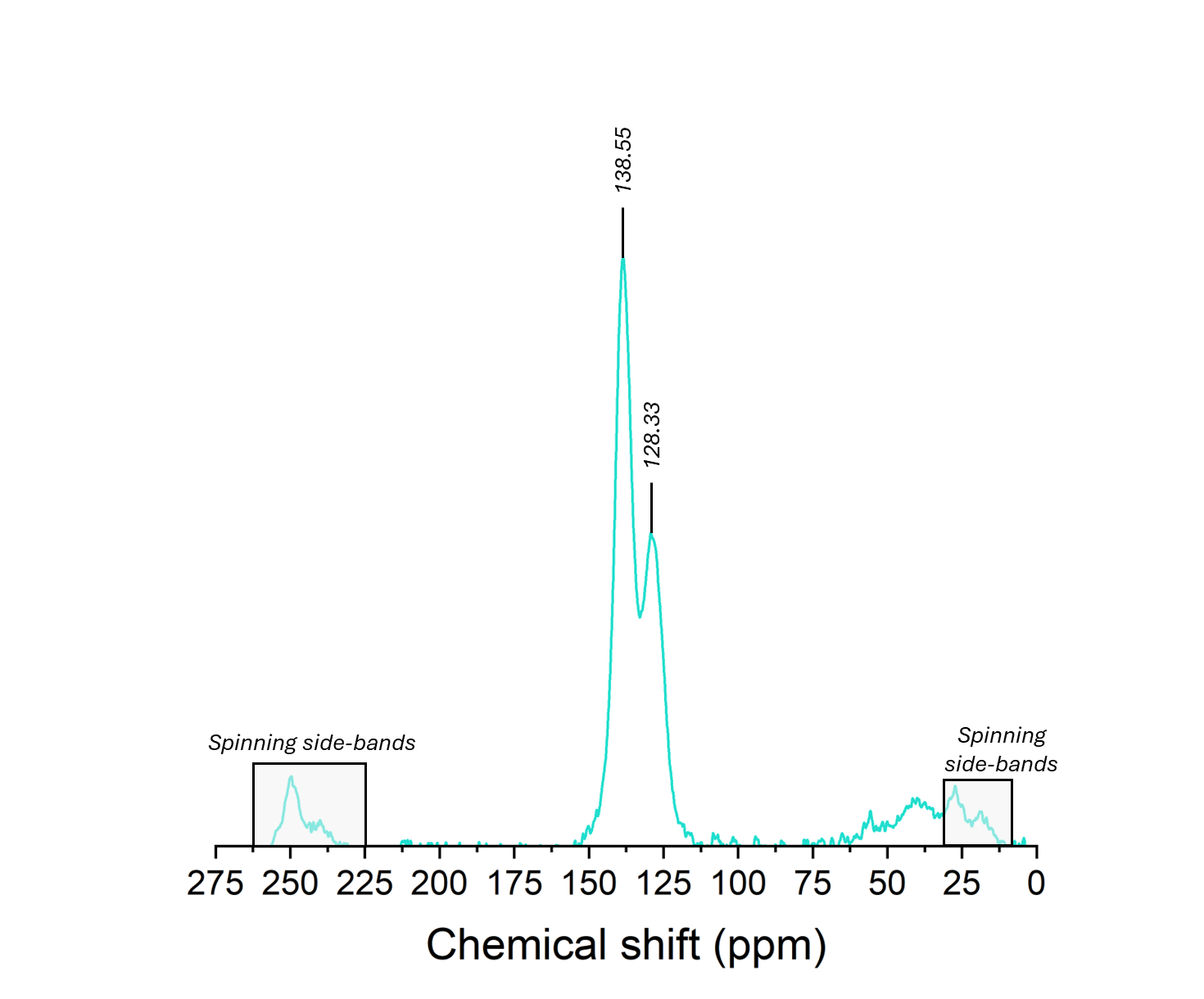


Figure S8. ^13^C NMR of BM-5, expanded to show spinning sidebands.

Figure S9. Potential Pummerer rearrangement mechanism and the product structure that would form, should this reaction take place.


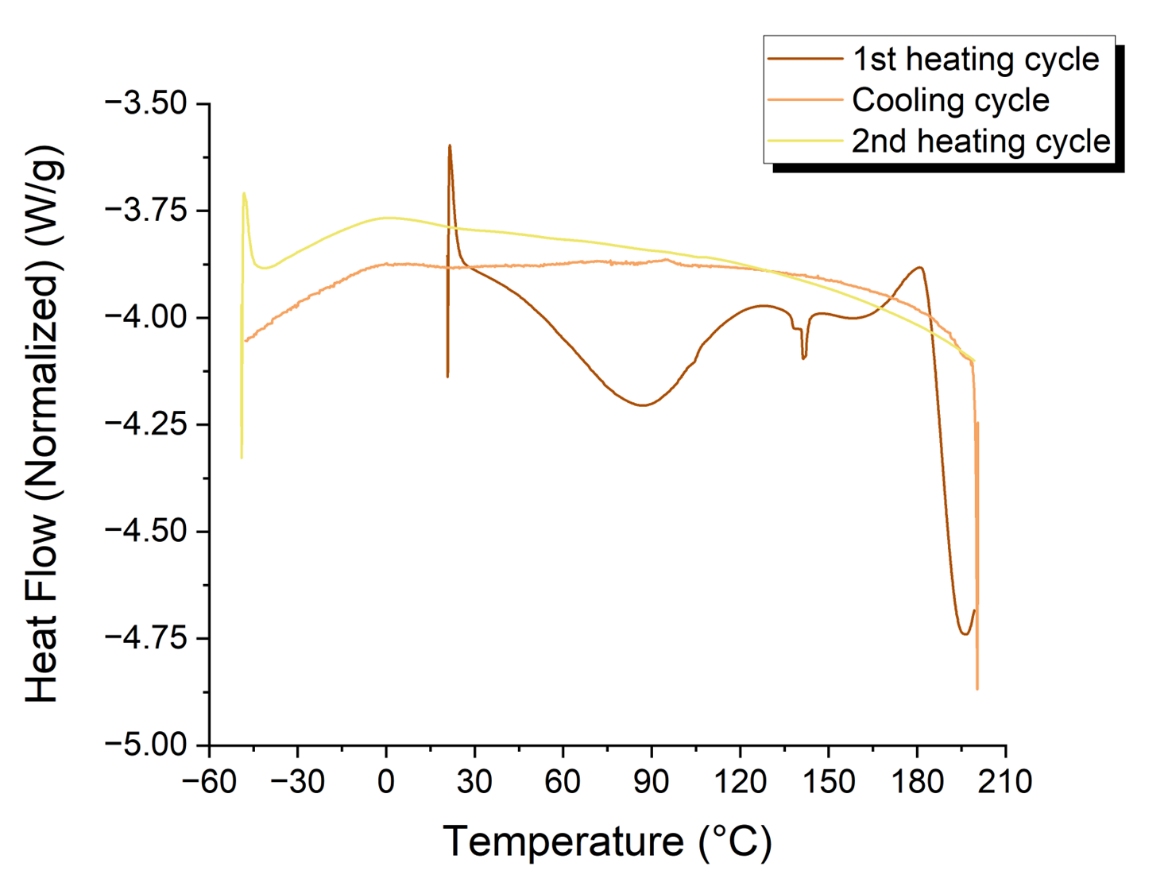


Figure S10. Heat-cool-heat DSC cycle of BM-1.


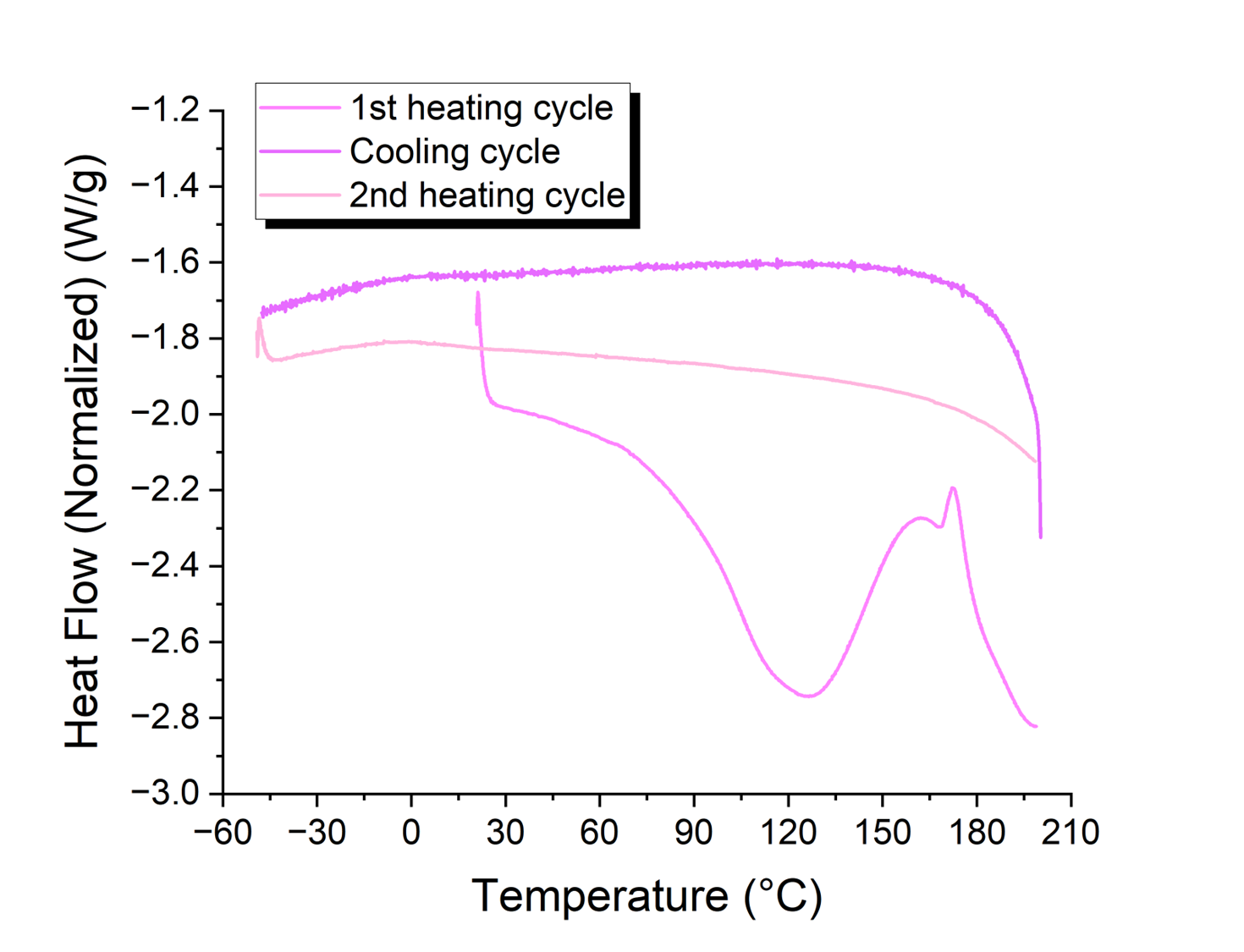


Figure S11. Heat-cool-heat DSC cycle of BM-3.


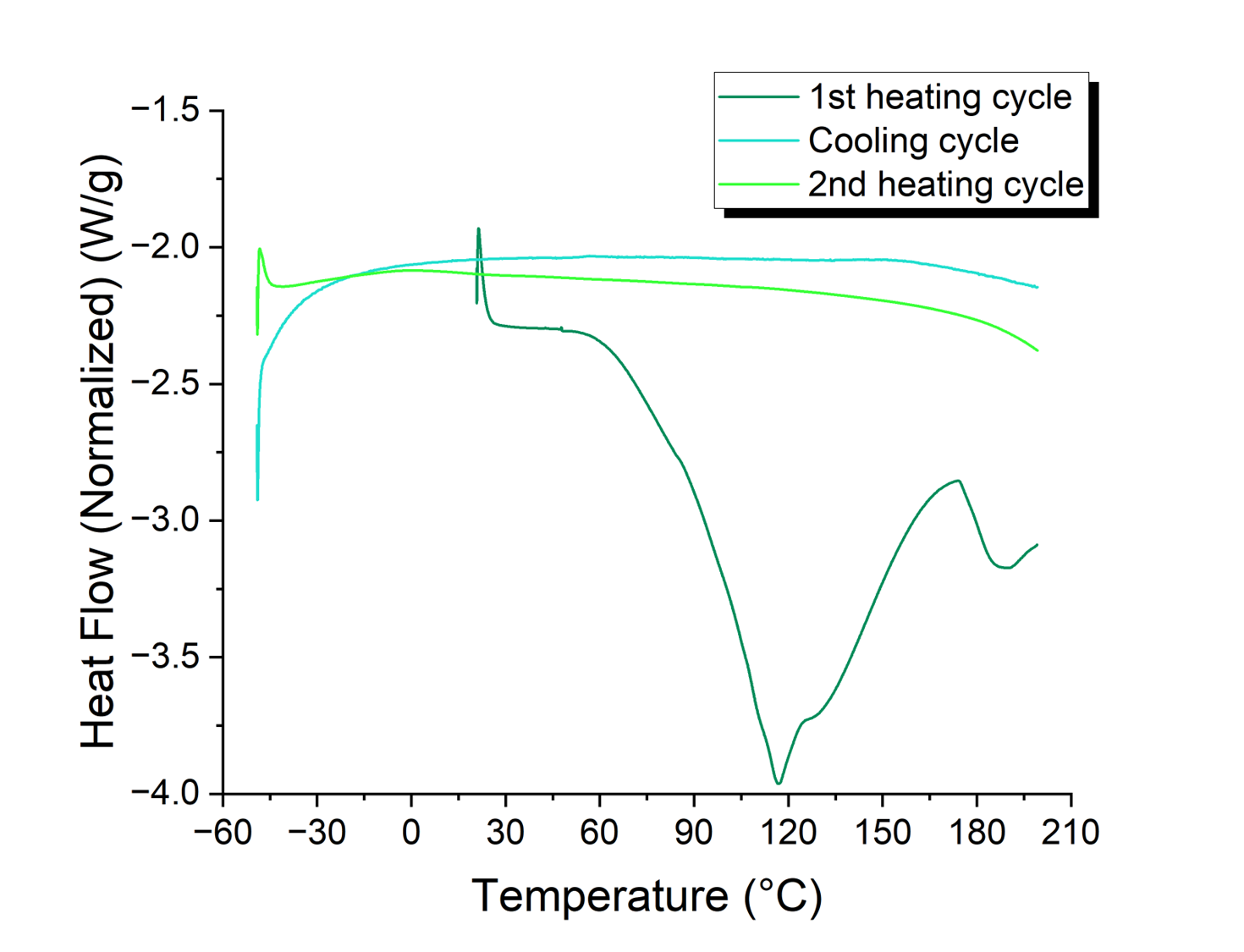


Figure S12. Heat-cool-heat DSC cycle of BM-5.

Table S1. Elemental CHNS analysis of BM-1, BM-3, and BM-5, and the average mass used in each measurement.

| Sample | C (wt.%) | H (wt.%) | N (wt.%) | S (wt.%) | Total mass (mg) |
| --- | --- | --- | --- | --- | --- |
| BM-1 | 70.65 ± 0.29 | 4.93 ± 0.02 | 0.07 ± 0.00 | 5.65 ± 0.15 | 1.34 ± 0.14 |
| BM-3 | 58.08 ± 2.32 | 4.93 ± 0.02 | 0.24 ± 0.02 | 14.29 ± 0.64 | 1.78 ± 0.25 |
| BM-5 | 46.33 ± 1.02 | 4.63 ± 0.35 | 0.06 ± 0.01 | 17.88 ± 0.39 | 1.55 ± 0.13 |


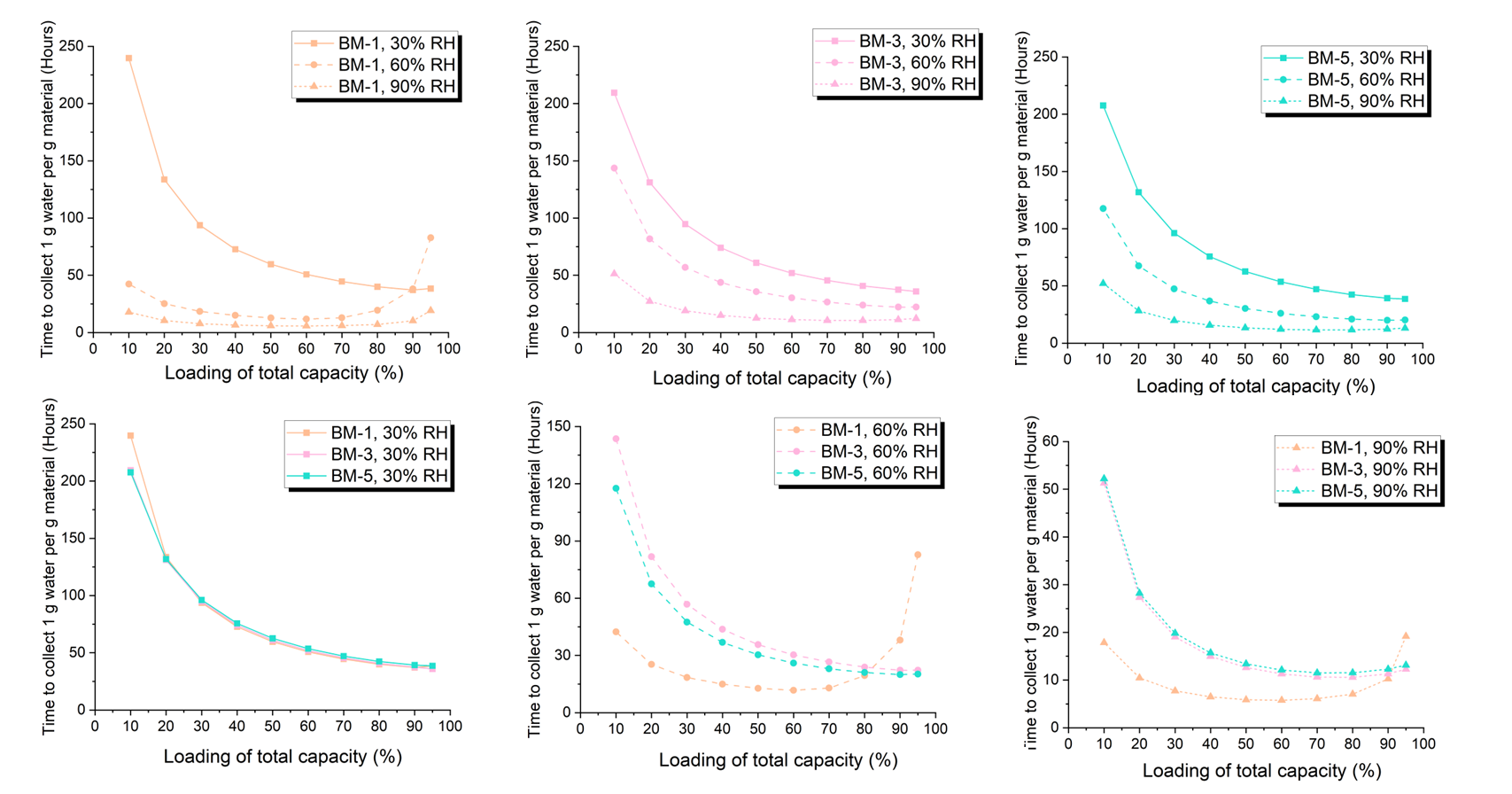


Figure S13. The calculated times to collect 1 g of water per g of material. Top row: Comparison of each HCP at different RH. Bottom row: Comparison of different HCPs at the same RH. BM-1 in orange, BM-3 in pink, BM-5 in green.

**Table S2.** The calculated number of cycles and time taken for each material to collect 1 g of water per g of material at 30, 60, and 90% RH. All cycles rounded up to the nearest full cycle. The fastest cycle in each case is underlined in bold.

|  | Time taken for **BM-1** to collect 1 g of water per g of material (hours) | | | Time taken for **BM-3** to collect 1 g of water per g of material (hours) | | | Time taken for **BM-5** to collect 1 g of water per g of material (hours) | | |
| --- | --- | --- | --- | --- | --- | --- | --- | --- | --- |
| % of total capacity | RH = 30% | RH = 60% | RH = 90% | RH = 30% | RH = 60% | RH = 90% | RH = 30% | RH = 60% | RH = 90% |
| 95 | 38.4 | 82.8 | 19.2 | **35.9** | 22.3 | 12.3 | **38.6** | 20.2 | 13.1 |
| 90 | **37.2** | 38.0 | 10.2 | 37.4 | **22.2** | 11.3 | 39.3 | **20.0** | 12.3 |
| 80 | 40.0 | 19.4 | 7.0 | 40.7 | 23.9 | **10.6** | 42.4 | 21.0 | **11.5** |
| 70 | 44.5 | 12.9 | 6.1 | 45.5 | 26.6 | **10.6** | 47.1 | 23.0 | **11.5** |
| 60 | 50.8 | **11.8** | **5.8** | 51.9 | 30.3 | 11.3 | 53.6 | 26.0 | 12.1 |
| 50 | 59.6 | 12.8 | 5.9 | 60.9 | 35.7 | 12.6 | 62.6 | 30.4 | 13.4 |
| 40 | 72.7 | 15.0 | 6.5 | 74.1 | 43.7 | 15.0 | 75.7 | 36.9 | 15.7 |
| 30 | 93.8 | 18.5 | 7.7 | 94.7 | 56.9 | 19.0 | 96.1 | 47.5 | 19.8 |
| 20 | 133.7 | 25.3 | 10.5 | 131.2 | 81.8 | 27.3 | 131.9 | 67.5 | 28.2 |
| 10 | 239.7 | 42.4 | 17.8 | 209.4 | 143.6 | 51.3 | 207.6 | 117.6 | 52.2 |

Table S3. Calculated time to collect 1 g of water per g of material of BM-1. Ads % relates to the % of max capacity at a designated RH. Ads time and des time relate to the adsorption and desorption times for the Ads %, as detailed in the main text. All other values calculated in accordance with equations S1-4 above).

| **% of max** | **Humidity** | **Ads %** | **ads time** | **des time** | **Cycle time mins** | **Cycle time hours** | **Mass of water per cycle** | **Cycles to get 1 g** | **Time to get 1 g water** |
| --- | --- | --- | --- | --- | --- | --- | --- | --- | --- |
| 95 | 30 | 1.83407 | 8.1 | 34.18 | 42.28 | 0.704666667 | 0.0183407 | 54.52354599 | 38.42092541 |
|  | 60 | 4.75418 | 218.56 | 17.63 | 236.19 | 3.9365 | 0.0475418 | 21.03412155 | 82.80081949 |
|  | 90 | 19.17433 | 194.08 | 26.59 | 220.67 | 3.677833333 | 0.19174325 | 5.215307449 | 19.18103158 |
| 90 | 30 | 1.73754 | 4.81 | 33.93 | 38.74 | 0.645666667 | 0.0173754 | 57.55263188 | 37.15981599 |
|  | 60 | 4.50396 | 85.31 | 17.46 | 102.77 | 1.712833333 | 0.0450396 | 22.20268386 | 38.02949701 |
|  | 90 | 18.16515 | 85.31 | 26.25 | 111.56 | 1.859333333 | 0.1816515 | 5.505046752 | 10.23571693 |
| 80 | 30 | 1.54448 | 3.38 | 33.67 | 37.05 | 0.6175 | 0.0154448 | 64.74671087 | 39.98109396 |
|  | 60 | 4.00352 | 29.53 | 17.13 | 46.66 | 0.777666667 | 0.0400352 | 24.97801934 | 19.42457304 |
|  | 90 | 16.1468 | 42.35 | 25.83 | 68.18 | 1.136333333 | 0.161468 | 6.193177596 | 7.037514141 |
| 70 | 30 | 1.35142 | 2.79 | 33.33 | 36.12 | 0.602 | 0.0135142 | 73.99624099 | 44.54573708 |
|  | 60 | 3.50308 | 10.29 | 16.79 | 27.08 | 0.451333333 | 0.0350308 | 28.54630782 | 12.88390026 |
|  | 90 | 14.12845 | 26.4 | 25.41 | 51.81 | 0.8635 | 0.1412845 | 7.077917252 | 6.111781547 |
| 60 | 30 | 1.15836 | 2.37 | 32.91 | 35.28 | 0.588 | 0.0115836 | 86.32894782 | 50.76142132 |
|  | 60 | 3.00264 | 4.72 | 16.45 | 21.17 | 0.352833333 | 0.0300264 | 33.30402579 | 11.75077043 |
|  | 90 | 12.1101 | 16.87 | 24.98 | 41.85 | 0.6975 | 0.121101 | 8.257570127 | 5.759655164 |
| 50 | 30 | 0.9653 | 2.03 | 32.49 | 34.52 | 0.575333333 | 0.009653 | 103.5947374 | 59.60150558 |
|  | 60 | 2.5022 | 3.12 | 16.03 | 19.15 | 0.319166667 | 0.025022 | 39.96483095 | 12.75544188 |
|  | 90 | 10.09175 | 11.21 | 24.39 | 35.6 | 0.593333333 | 0.1009175 | 9.909084153 | 5.879389931 |
| 40 | 30 | 0.77224 | 1.78 | 31.9 | 33.68 | 0.561333333 | 0.0077224 | 129.4934217 | 72.68897407 |
|  | 60 | 2.00176 | 2.36 | 15.61 | 17.97 | 0.2995 | 0.0200176 | 49.95603869 | 14.96183359 |
|  | 90 | 8.0734 | 7.5 | 23.89 | 31.39 | 0.523166667 | 0.080734 | 12.38635519 | 6.480128157 |
| 30 | 30 | 0.57918 | 1.52 | 31.06 | 32.58 | 0.543 | 0.0057918 | 172.6578956 | 93.75323734 |
|  | 60 | 1.50132 | 1.77 | 14.85 | 16.62 | 0.277 | 0.0150132 | 66.60805158 | 18.45043029 |
|  | 90 | 6.05505 | 4.97 | 23.13 | 28.1 | 0.468333333 | 0.0605505 | 16.51514025 | 7.734590686 |
| 20 | 30 | 0.38612 | 1.27 | 29.7 | 30.97 | 0.516166667 | 0.0038612 | 258.9868435 | 133.6803757 |
|  | 60 | 1.00088 | 1.43 | 13.75 | 15.18 | 0.253 | 0.0100088 | 99.91207737 | 25.27775558 |
|  | 90 | 4.0367 | 3.28 | 22.03 | 25.31 | 0.421833333 | 0.040367 | 24.77271038 | 10.449955 |
| 10 | 30 | 0.19306 | 1.02 | 26.75 | 27.77 | 0.462833333 | 0.0019306 | 517.9736869 | 239.7354881 |
|  | 60 | 0.50044 | 1.09 | 11.64 | 12.73 | 0.212166667 | 0.0050044 | 199.8241547 | 42.39602483 |
|  | 90 | 2.01835 | 1.94 | 19.67 | 21.61 | 0.360166667 | 0.0201835 | 49.54542076 | 17.84460905 |

Table S4. Calculated time to collect 1 g of water per g of material of BM-3. Ads % relates to the % of max capacity at a designated RH. Ads time and des time relate to the adsorption and desorption times for the Ads %, as detailed in the main text. All other values calculated in accordance with equations S1-4 above).

| **% of max** | **Humidity** | **Ads %** | **ads time** | **des time** | **Cycle time mins** | **Cycle time hours** | **Mass of water per cycle** | **Cycles to get 1 g** | **Time to get 1 g water** |
| --- | --- | --- | --- | --- | --- | --- | --- | --- | --- |
| 95 | 30 | 21.5517 | 50.88 | 412.91 | 463.79 | 7.729833333 | 0.215517 | 4.640005197 | 35.86646684 |
|  | 60 | 41.67774 | 86.49 | 470.62 | 557.11 | 9.285166667 | 0.41677735 | 2.399362633 | 22.27848194 |
|  | 90 | 110.9849 | 400.42 | 417.96 | 818.38 | 13.63966667 | 1.1098489 | 0.901023554 | 12.28966093 |
| 90 | 30 | 20.04174 | 38.73 | 411.22 | 449.95 | 7.499166667 | 0.2004174 | 4.989586732 | 37.4177425 |
|  | 60 | 39.48417 | 57.29 | 469.27 | 526.56 | 8.776 | 0.3948417 | 2.532660557 | 22.22662905 |
|  | 90 | 105.1436 | 297.63 | 415.67 | 713.3 | 11.88833333 | 1.0514358 | 0.951080418 | 11.30676103 |
| 80 | 30 | 17.81488 | 26.92 | 408.27 | 435.19 | 7.253166667 | 0.1781488 | 5.613285074 | 40.71409219 |
|  | 60 | 35.09704 | 36.96 | 466.49 | 503.45 | 8.390833333 | 0.3509704 | 2.849243127 | 23.90752421 |
|  | 90 | 93.46096 | 181.69 | 411.12 | 592.81 | 9.880166667 | 0.9346096 | 1.06996547 | 10.57143717 |
| 70 | 30 | 15.58802 | 20.42 | 404.82 | 425.24 | 7.087333333 | 0.1558802 | 6.415182942 | 45.4665399 |
|  | 60 | 30.70991 | 25.99 | 464.12 | 490.11 | 8.1685 | 0.3070991 | 3.256277859 | 26.5989057 |
|  | 90 | 81.77834 | 115.53 | 406.16 | 521.69 | 8.694833333 | 0.8177834 | 1.22281768 | 10.63219593 |
| 60 | 30 | 13.36116 | 15.94 | 400.43 | 416.37 | 6.9395 | 0.1336116 | 7.484380099 | 51.9378557 |
|  | 60 | 26.32278 | 18.9 | 459.82 | 478.72 | 7.978666667 | 0.2632278 | 3.798990836 | 30.31088155 |
|  | 90 | 70.09572 | 74.01 | 401.16 | 475.17 | 7.9195 | 0.7009572 | 1.426620627 | 11.29812205 |
| 50 | 30 | 11.13443 | 12.48 | 394.6 | 407.08 | 6.784666667 | 0.1113443 | 8.981151258 | 60.93411757 |
|  | 60 | 21.93565 | 13.95 | 455.52 | 469.47 | 7.8245 | 0.2193565 | 4.558789003 | 35.67024456 |
|  | 90 | 58.4131 | 46.85 | 395.68 | 442.53 | 7.3755 | 0.584131 | 1.711944752 | 12.62644852 |
| 40 | 30 | 8.90744 | 9.7 | 386.25 | 395.95 | 6.599166667 | 0.0890744 | 11.22657015 | 74.0860075 |
|  | 60 | 17.54852 | 10.29 | 449.86 | 460.15 | 7.669166667 | 0.1754852 | 5.698486254 | 43.70264083 |
|  | 90 | 46.73048 | 30.38 | 389.68 | 420.06 | 7.001 | 0.4673048 | 2.13993094 | 14.98165651 |
| 30 | 30 | 6.68058 | 7.34 | 372.41 | 379.75 | 6.329166667 | 0.0668058 | 14.9687602 | 94.73977808 |
|  | 60 | 13.16139 | 7.42 | 441.51 | 448.93 | 7.482166667 | 0.1316139 | 7.597981672 | 56.8493652 |
|  | 90 | 35.04786 | 17.56 | 382.59 | 400.15 | 6.669166667 | 0.3504786 | 2.853241254 | 19.02874146 |
| 20 | 30 | 4.45372 | 5.15 | 345.32 | 350.47 | 5.841166667 | 0.0445372 | 22.4531403 | 131.1525347 |
|  | 60 | 8.77426 | 5.15 | 425.41 | 430.56 | 7.176 | 0.0877426 | 11.39697251 | 81.78467472 |
|  | 90 | 23.36524 | 9.96 | 373.06 | 383.02 | 6.383666667 | 0.2336524 | 4.27986188 | 27.32121162 |
| 10 | 30 | 2.22686 | 3.12 | 276.72 | 279.84 | 4.664 | 0.0222686 | 44.90628059 | 209.4428927 |
|  | 60 | 4.38713 | 3.12 | 374.86 | 377.98 | 6.299666667 | 0.0438713 | 22.79394502 | 143.5942556 |
|  | 90 | 11.68262 | 5.06 | 354.59 | 359.65 | 5.994166667 | 0.1168262 | 8.559723761 | 51.30841084 |

Table S5. Calculated time to collect 1 g of water per g of material of BM-5. Ads % relates to the % of max capacity at a designated RH. Ads time and des time relate to the adsorption and desorption times for the Ads %, as detailed in the main text. All other values calculated in accordance with equations S1-4 above).

| **% of max** | **Humidity** | **Ads %** | **ads time** | **des time** | **Cycle time mins** | **Cycle time hours** | **Mass of water per cycle** | **Cycles to get 1 g** | **Time to get 1 g water** |
| --- | --- | --- | --- | --- | --- | --- | --- | --- | --- |
| 95 | 30 | 25.92341 | 79 | 521.25 | 600.25 | 10.00416667 | 0.2592341 | 3.857517202 | 38.591245 |
|  | 60 | 52.16507 | 130.38 | 502.01 | 632.39 | 10.53983333 | 0.5216507 | 1.916991581 | 20.20477176 |
|  | 90 | 136.9917 | 534.4 | 545.47 | 1079.87 | 17.99783333 | 1.3699171 | 0.729971179 | 13.13789961 |
| 90 | 30 | 24.55902 | 59.51 | 519.22 | 578.73 | 9.6455 | 0.2455902 | 4.071823713 | 39.27477562 |
|  | 60 | 49.41954 | 93.25 | 499.99 | 593.24 | 9.887333333 | 0.4941954 | 2.023491113 | 20.00693113 |
|  | 90 | 129.7816 | 415.93 | 541.84 | 957.77 | 15.96283333 | 1.2978162 | 0.770525133 | 12.29976428 |
| 80 | 30 | 21.83024 | 40.93 | 514.75 | 555.68 | 9.261333333 | 0.2183024 | 4.580801677 | 42.42433126 |
|  | 60 | 43.92848 | 58.82 | 495.69 | 554.51 | 9.241833333 | 0.4392848 | 2.276427502 | 21.03836357 |
|  | 90 | 115.3614 | 263.61 | 534.33 | 797.94 | 13.299 | 1.1536144 | 0.866840775 | 11.52811546 |
| 70 | 30 | 19.10146 | 30.64 | 509.52 | 540.16 | 9.002666667 | 0.1910146 | 5.235201917 | 47.13077779 |
|  | 60 | 38.43742 | 40.68 | 490.88 | 531.56 | 8.859333333 | 0.3843742 | 2.601631431 | 23.04872006 |
|  | 90 | 100.9413 | 170.46 | 526.64 | 697.1 | 11.61833333 | 1.0094126 | 0.990675171 | 11.50999436 |
| 60 | 30 | 16.37268 | 23.64 | 502.85 | 526.49 | 8.774833333 | 0.1637268 | 6.107735569 | 53.59436166 |
|  | 60 | 32.94636 | 29.2 | 485.31 | 514.51 | 8.575166667 | 0.3294636 | 3.03523667 | 26.02766031 |
|  | 90 | 86.52108 | 110.03 | 518.46 | 628.49 | 10.47483333 | 0.8652108 | 1.155787699 | 12.10668352 |
| 50 | 30 | 13.6439 | 18.32 | 494.16 | 512.48 | 8.541333333 | 0.136439 | 7.329282683 | 62.60184649 |
|  | 60 | 27.4553 | 21.18 | 478.81 | 499.99 | 8.333166667 | 0.274553 | 3.642284003 | 30.35175965 |
|  | 90 | 72.1009 | 69.86 | 509.77 | 579.63 | 9.6605 | 0.721009 | 1.386945239 | 13.39858448 |
| 40 | 30 | 10.91512 | 14.1 | 481.68 | 495.78 | 8.263 | 0.1091512 | 9.161603354 | 75.70232851 |
|  | 60 | 21.96424 | 15.27 | 470.46 | 485.73 | 8.0955 | 0.2196424 | 4.552855004 | 36.85763769 |
|  | 90 | 57.68072 | 43.11 | 500.15 | 543.26 | 9.054333333 | 0.5768072 | 1.733681549 | 15.69733064 |
| 30 | 30 | 8.18634 | 10.3 | 461.93 | 472.23 | 7.8705 | 0.0818634 | 12.21547114 | 96.1418656 |
|  | 60 | 16.47318 | 10.8 | 458.56 | 469.36 | 7.822666667 | 0.1647318 | 6.070473339 | 47.48728944 |
|  | 90 | 43.26054 | 25.56 | 488.84 | 514.4 | 8.573333333 | 0.4326054 | 2.311575399 | 19.81790642 |
| 20 | 30 | 5.45756 | 7.09 | 424.8 | 431.89 | 7.198166667 | 0.0545756 | 18.32320671 | 131.8934958 |
|  | 60 | 10.98212 | 7.17 | 437.8 | 444.97 | 7.416166667 | 0.1098212 | 9.105710009 | 67.52946304 |
|  | 90 | 28.84036 | 14.08 | 473.82 | 487.9 | 8.131666667 | 0.2884036 | 3.467363098 | 28.19544093 |
| 10 | 30 | 2.72878 | 4.05 | 335.86 | 339.91 | 5.665166667 | 0.0272878 | 36.64641342 | 207.6080397 |
|  | 60 | 5.49106 | 4.05 | 383.37 | 387.42 | 6.457 | 0.0549106 | 18.21142002 | 117.5911391 |
|  | 90 | 14.42018 | 6.83 | 444.88 | 451.71 | 7.5285 | 0.1442018 | 6.934726196 | 52.20808617 |


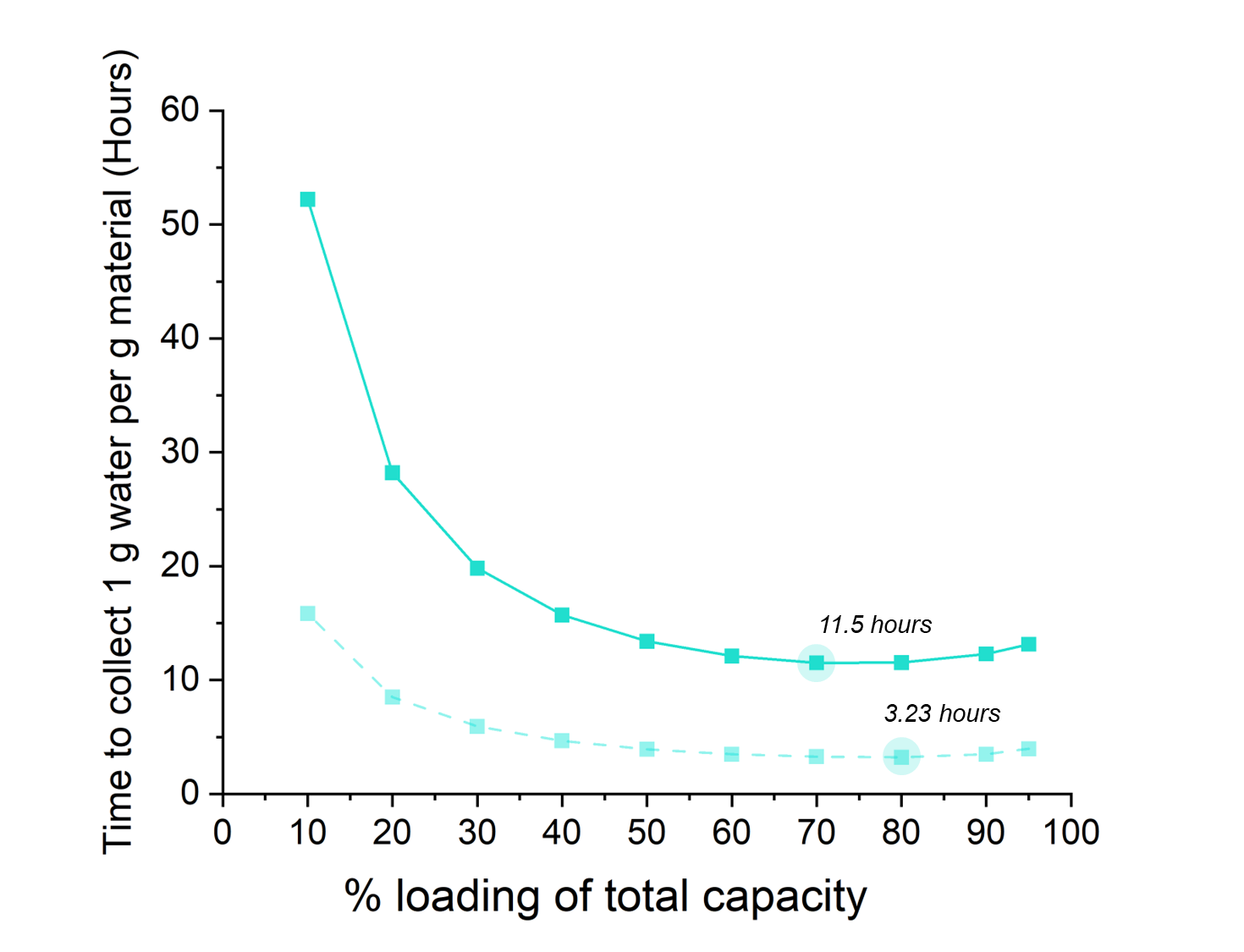


Figure S14. Comparison of the 1 g g^-1^ collection rates of BM-5 at 25 °C (solid line) and 45 °C (dashed line) at 90% RH. Optimum charging levels highlighted in circles.


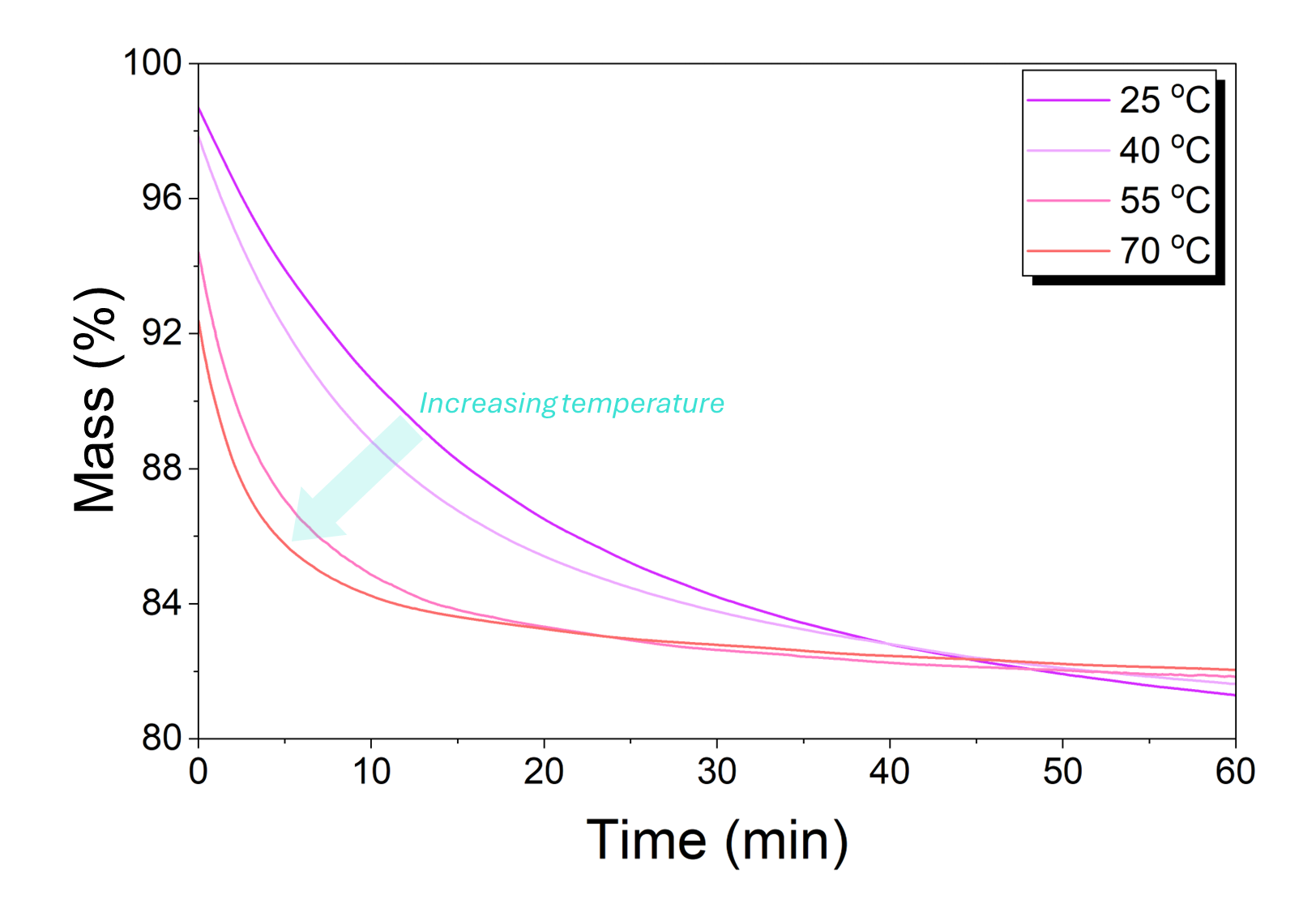


Figure S15. The desorption rate of BM-5 after conditioning at 75% RH and 20-23 °C, measured by TGA isothermal hold at increasing temperature.


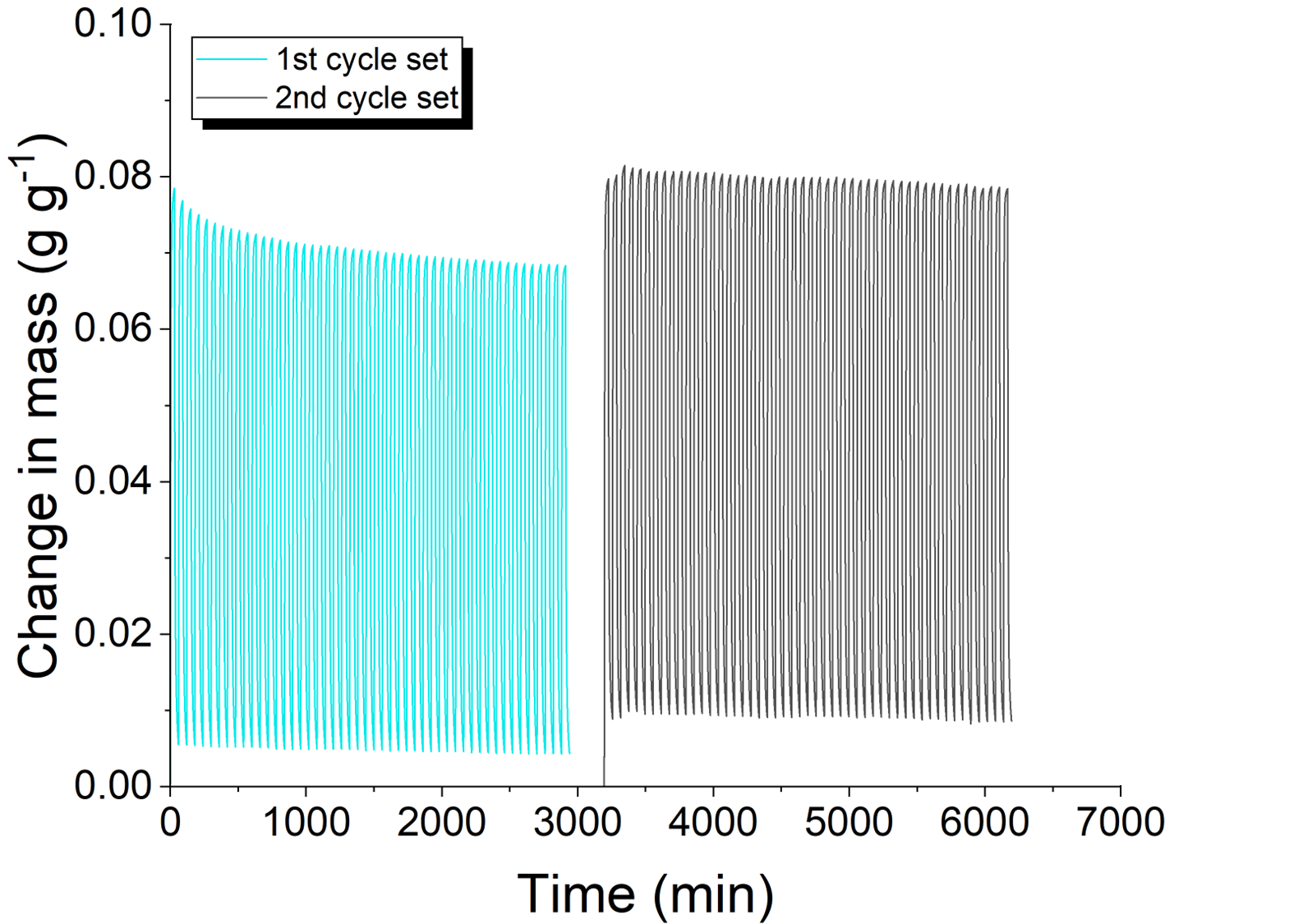


Figure S16. Cycling of BM-5 for 30 min at 0 and 40% RH (25 °C) over 60 cycles. The capacity is observed to decrease. After the measurement the sample was removed from the DVS and dried for 24 hours in air at room temperature. The cycling was repeated and the capacity recovered. The capacity is lower than other cycling measurements due to the reduced time of each cycle (30 min at 40% RH, 30 min at 0% RH).


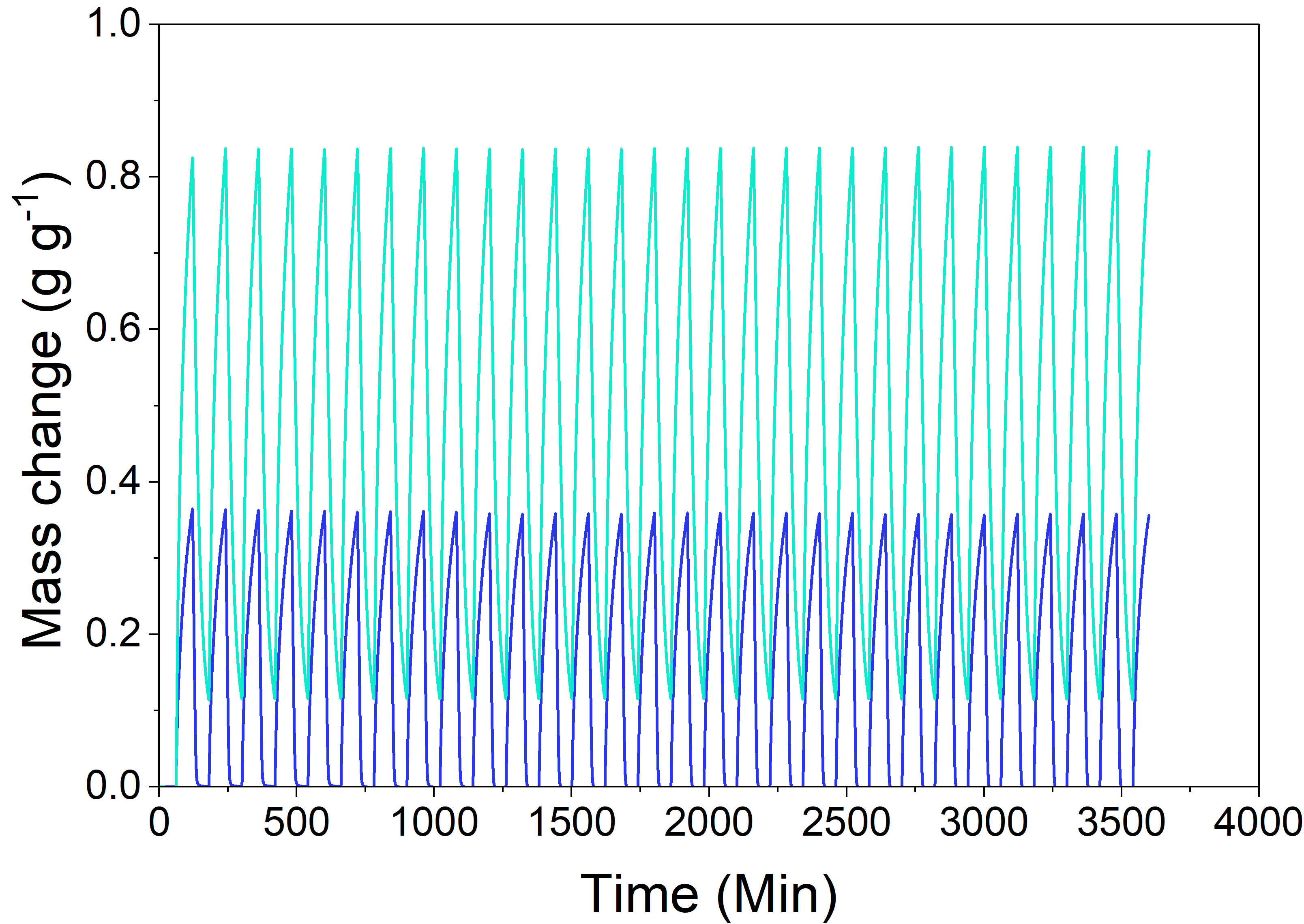


Figure S17. Cycling of BM-5 over 30 cycles at 40% RH (blue) and 90% RH (green) demonstrating the initial stability of BM-5 to cycle in high humidity environments.

Table S6. A comparison of the swelling percentage of the reported polymers. Due to low yield the swelling of BM-1 could not be determined in triplicate.

| **Sample** | **Swelling percentage (%)** |
| --- | --- |
| BM-1 | 100 |
| BM-3 | 122 ± 3 |
| BM-5 | 149 ± 33 |

Table S7. A comparison of 30 previously reported AWH materials with our BM-X series.

| **Material** | **Network chemistry** | **BET surface area [m^2^/g]** | **H_2_O uptake at 0.3 P/P_0_ [g/g]** | **Total uptake capacity [g/g]** | **Uptake T [°C]** | **Ref.** |
| --- | --- | --- | --- | --- | --- | --- |
| **Metal-organic Frameworks** | | | | | | |
| Cr-MIL-101 | Cr, terephthalic acid | 2500 | 0.09 | 0.86 | 25 | ^[[1]](#endnote-1)^ |
| MOF-801 | Zr, fumaric acid | 990 | 0.26 | 0.36 | 25 | ^[[2]](#endnote-2)^ |
| CAU-10 | Al, 1,3-benzene dicarboxylic acid | 635 | 0.29 | 0.36 | 25 | ^[[3]](#endnote-3)^ |
| MIL-160 | Al, 2,5-furandicarboxylic acid | 1070 | 0.34 | 0.36 | 25 | ^[[4]](#endnote-4)^ |
| MOF-303 | Al, 1-H-pyrazole-3,5-dicarboxylate | 1280 | 0.40 | 0.44 | 25 | ^[[5]](#endnote-5)^ |
| MOF-LA2–1 | Al, (E)-5-(2-carboxyvinyl)-1H-pyrazole-3-carboxylic acid | 1892 | 0.58 | 0.64 | 25 | ^[[6]](#endnote-6)^ |
| **Covalent Organic Frameworks** | | | | | | |
| TpBD | Ketoenamine | 341 | 0.02 | 0.15 | 25 | ^[[7]](#endnote-7)^ |
| PI-3-COF | Imine / Triazine | 1340 | 0.03 | 0.46 | 25 | ^[[8]](#endnote-8)^ |
| Py-HMPA | Imine / Pyrene / –OH | 705 | 0.07 | 0.23 | 25 | ^[[9]](#endnote-9)^ |
| ATFG-COF | Keto-Enol tautomer | 520 | 0.14 | 0.25 | 25 | ^[[10]](#endnote-10)^ |
| 3D-CageCOF-1 | Imine / Phenyl ether | 1040 | 0.16 | 0.30 | 25 | ^[[11]](#endnote-11)^ |
| COF-SO_3_H | Ketoenamine / –SO_3_H | 280 | 0.19 | 0.31 | 25 | ^[[12]](#endnote-12)^ |
| COF-ok | Ketoenamine / Pyrene | 1194 | 0.20 | 0.64 | 25 | ^[[13]](#endnote-13)^ |
| TB-COF-Li | Ketoenamine / –SO_3_H / LiCl | 439 | 0.24 | 0.44 | 25 | ^[[14]](#endnote-14)^ |
| COF-480-hydrazide | Hydrazide | 989 | 0.32 | 0.44 | 25 | ^[[15]](#endnote-15)^ |
| g-DZPH-COF | Vinylene / Pyridazine | 960 | 0.39 | 1.00 | 25 | ^[[16]](#endnote-16)^ |
| DAAQ-TFP-SO3H@LiCl | Ketoenamine / –SO_3_H / LiCl | 635 | 0.46 | 1.21 | 25 | ^[[17]](#endnote-17)^ |
| DHTA-Pa | (Ketoenamine)_2_ / Enamine | 2099 | 0.48 | 0.65 | 25 | ^[[18]](#endnote-18)^ |
| COF-309 | Imine / Ketoenamine | 1698 | 0.58 | 0.74 | 25 | ^[[19]](#endnote-19)^ |
| **Covalent Triazine Frameworks** | | | | | | |
| CTF-TPC | Triptycene | 1668 | 0.07 | 0.39 | 20 | ^[[20]](#endnote-20)^ |
| pym-CTF500 | Pyrimidine | 208 | 0.13 | 0.23 | 25 | ^[[21]](#endnote-21)^ |
| FJU-CTF-FIZ500 | Benzimidazole | 2042 | 0.21 | 0.82 | 23 | ^[[22]](#endnote-22)^ |
| bpim-CTF-400 | Bis(pyridyl) imidazolium | 786 | 0.22 | 0.38 | 25 | ^[[23]](#endnote-23)^ |
| **Activated carbon-based materials** | | | | | | |
| AC-K_2_CO_3_-900 | AC / K_2_CO_3_ | 1946 | 0.02 | 0.80 | 25 | ^[[24]](#endnote-24)^ |
| O-NPS | AC / Various oxygen species | 484 | 0.05 | 0.18 | 25 | ^[[25]](#endnote-25)^ |
| Bio-NPS500 | Biobased AC | 934 | 0.10 | 0.30 | 25 | ^[[26]](#endnote-26)^ |
| SAC-200 | Powderous AC / –SO_3_H | 252 | 0.28 | 1.43 | 25 | ^[[27]](#endnote-27)^ |
| **Amorphous micro-/mesoporous POPs** | | | | | | |
| OHCP-60 | Thermal oxidation | 462 | 0.06 | 0.35 | 25 | ^[[28]](#endnote-28)^ |
| 3D ep-POP | Oxirane (epoxide) | 779 | 0.18 | 0.41 | 25 | ^[[29]](#endnote-29)^ |
| SHCP-10 | –SO_3_H | 697 | 0.22 | 0.81 | 25 | ^[[30]](#endnote-30)^ |
| BM-1 | S-S, –SO_3_H | 0 | 0.03 | 0.22 | 25 | This work |
| BM-3 | S-S, –SO_3_H | 0 | 0.23 | 1.24 | 25 | This work |
| BM-5 | S-S, –SO_3_H | 41 | 0.26 | 1.39 (1.60 after cycling) | 25 | This work |

1. J. Canivet, J. Bonnefoy, C. Daniel, A. Legrand, B. Coasne, D. Farrusseng, *New J. Chem.,* 2014, **38 (7)**, 3102–3111. [↑](#endnote-ref-1)
2. H. Furukawa, F. Gándara, Y. B. Zhang, J. Jiang, W. L. Queen, M. R. Hudson, O. M. Yaghi, O. M. *J. Am. Chem. Soc.,* 2014, **136 (11)**, 4369–4381. [↑](#endnote-ref-2)
3. H. Reinsch, M. A. van der Veen, B. Gil, B. Marszalek, T. Verbiest, D. de Vos, N. Stock, *Chem. Mater.,* 2013, **25 (1)**, 17–26. [↑](#endnote-ref-3)
4. A. Cadiau, J. S. Lee, D. Damasceno Borges, P. Fabry, T. Devic, M. T. Wharmby, C. Martineau, D. Foucher, F. Taulelle, C. H. Jun, Y. K. Hwang, N. Stock, M. F. De Lange, F. Kapteijn, J. Gascon, G. Maurin, J. S. Chang, C. Serre, *Adv. Mater.,*  2015, **27 (32)**, 4775–4780. [↑](#endnote-ref-4)
5. N. Hanikel, X. Pei, S. Chheda, H. Lyu, W. Jeong, J. Sauer, L. Gagliardi, O. M. Yaghi, *Science,* 2021, **374 (6566)**, 454–459. [↑](#endnote-ref-5)
6. N. Hanikel, D. Kurandina, S. Chheda, Z. Zheng, Z. Rong, S. E. Neumann, J. Sauer, J. I. Siepmann, L. Gagliardi, O. M. Yaghi, *ACS Cent. Sci.,* 2023, **9 (3)**, 551–557. [↑](#endnote-ref-6)
7. B. P. Biswal, S. Kandambeth, S. Chandra, D. B. Shinde, S. Bera, S. Karak, B. Garai, U. K. Kharul, R. Banerjee, *J. Mater. Chem. A,* 2015, **3 (47)**, 23664–23669. [↑](#endnote-ref-7)
8. L. Grunenberg, G. Savasci, S. T. Emmerling, F. Heck, S. Bette, A. Cima Bergesch, C. Ochsenfeld, B. V. Lotsch, *J. Am. Chem. Soc.,* 2023, **145 (24)**, 13241–13248. [↑](#endnote-ref-8)
9. Y. Liu, W. K. Han, W. Chi, J. X. Fu, Y. Mao, X. Yan, J. X. Shao, Y. Jiang, Z. G. Gu, *Appl. Catal. B.,* 2023, **338**, 123074. [↑](#endnote-ref-9)
10. L. Stegbauer, M. W. Hahn, A. Jentys, G. Savasci, C. Ochsenfeld, J. A. Lercher, B. V. Lotsch, *Chem. Mater.* 2015, **27 (23)**, 7874–7881. [↑](#endnote-ref-10)
11. Q. Zhu, X. Wang, R. Clowes, P. Cui, L. Chen, M. A. Little, A. I. Cooper, *J. Am. Chem. Soc.,* 2020, **142** **(39)**, 16842–16848. [↑](#endnote-ref-11)
12. P. Schweng, C. Li, P. Guggenberger, F. Kleitz, R. T. Woodward, *ChemSusChem*, 2024, **17 (20)**, e202301906. [↑](#endnote-ref-12)
13. L. H. Chen, W. K. Han, X. Yan, J. Zhang, Y. Jiang, Z. G. Gu, *ChemSusChem,* 2022, **15 (24)**, e202201824. [↑](#endnote-ref-13)
14. Z. Shi, Y. Guo, X. Zou, J. Zhang, Z. Chen, M. Shan, Z. Zhang, S. Guo, F. Yan, *Angew. Chem. Int. Ed.,* 2024, **64 (9)***,* e202420619. [↑](#endnote-ref-14)
15. H. L. Nguyen, C. Gropp, N. Hanikel, A. Möckel, A. Lund, O. M. Yaghi, *ACS Cent. Sci.,* 2022, **8 (7)**, 926–932. [↑](#endnote-ref-15)
16. K. Mou, F. Meng, Z. Zhang, X. Li, M. Li, Y. Jiao, Z. Wang, X. Bai, F. Zhang, *Angew. Chem. Int. Ed.,* 2024, **63 (34)**, e202402446. [↑](#endnote-ref-16)
17. Y. Liu, Y. Zhu, Q. Mao, W. Chen, *Small,* 2024, **20 (51)**, 2406803. [↑](#endnote-ref-17)
18. C. Sun, Y. Zhu, P. Shao, L. Chen, X. Huang, S. Zhao, D. Ma, X. Jing, B. Wang, X. Feng, *Angew. Chem. Int. Ed.,* 2023, **62 (11)**, e202217103. [↑](#endnote-ref-18)
19. H. L. Nguyen, A. Darù, S. Chheda, A. H. Alawadhi, S. E. Neumann, L. Wang, X. Bai, M. O. Alawad, C. Borgs, J. T. Chayes, J. Sauer, L. Gagliardi, O. M. Yaghi, *ACS Cent. Sci.,* 2025, **11 (5)**, 665–671. [↑](#endnote-ref-19)
20. S. Dey, A. Bhunia, D. Esquivel, C. Janiak, *J. Mater. Chem. A,* 2016, **4 (17)**, 6259–6263. [↑](#endnote-ref-20)
21. S. Hug, L. Stegbauer, H. Oh, M. Hirscher, B. V. Lotsch, *Chem. Mater.,* 2015, **27 (23)**, 8001–8010. [↑](#endnote-ref-21)
22. J. Huang, Y. Yang, L. Chen, Z. Zhang, S. Xiang, *ZAAC,* 2023, **649 (19)**, e202300167. [↑](#endnote-ref-22)
23. K. Park, K. Lee, H. Kim, V. Ganesan, K. Cho, S. K. Jeong, S. Yoon, *J. Mater. Chem. A,* 2017, **5 (18)**, 8576–8582. [↑](#endnote-ref-23)
24. T. Horikawa, Y. Kitakaze, T. Sekida, J. Hayashi, M. Katoh, *Bioresour. Technol.,* 2010, **101 (11)**, 3964–3969. [↑](#endnote-ref-24)
25. U. Legrand, D. Klassen, S. Watson, A. Aufoujal, B. Nisol, R. Boudreault, K. E. Waters, J. L. Meunier, P. L. Girard-Lauriault, M. R. Wertheimer, J. R. Tavares, *Ind. Eng. Chem. Res.,* 2021, **60 (35)**, 12923–12933. [↑](#endnote-ref-25)
26. S. Ponton, T. Salvi, P. L. Girard-Lauriault, J. R. Tavares, *ACS Sustain. Chem. Eng.,* 2025, **13 (9)**, 3500–3511. [↑](#endnote-ref-26)
27. P. Schweng, A. Naryshkina, R. A. Glabonjat, R. T. Woodward, *Carbon*, 2026, **247**, 120964. [↑](#endnote-ref-27)
28. P. Schweng, L. Präg, R. T. Woodward, *ACS Appl. Mater. Interfaces,* 2024, **16 (43)**, 58566–58572. [↑](#endnote-ref-28)
29. Y. Byun, A. Coskun, *Angew. Chem. Int. Ed.,* 2018, **57 (12)**, 3173–3177. [↑](#endnote-ref-29)
30. P. Schweng, F. Mayer, D. Galehdari, K. Weiland, R. T. Woodward, *Small,* 2023, **19 (50)**, 2304562. [↑](#endnote-ref-30)
